# Comprehensive Analysis of ABA Effects on Ethylene Biosynthesis and Signaling during Tomato Fruit Ripening

**DOI:** 10.1371/journal.pone.0154072

**Published:** 2016-04-21

**Authors:** Wangshu Mou, Dongdong Li, Jianwen Bu, Yuanyuan Jiang, Zia Ullah Khan, Zisheng Luo, Linchun Mao, Tiejin Ying

**Affiliations:** 1 College of Biosystems Engineering and Food Science, Fuli Institute of Food Science, Zhejiang Key Laboratory for Agro-Food Processing, Zhejiang R & D Center for Food Technology and Equipment, Zhejiang University, Hangzhou 310058, People’s Republic of China; 2 Department of Food Science and Engineering, Shandong Agriculture and Engineering University, Ji’nan 250100, People’s Republic of China; 3 Department of Agriculture, Abdul Wali Khan University, Mardan 23200, KPK., Pakistan; Institute of Genetics and Developmental Biology, Chinese Academy of Sciences, CHINA

## Abstract

ABA has been widely acknowledged to regulate ethylene biosynthesis and signaling during fruit ripening, but the molecular mechanism underlying the interaction between these two hormones are largely unexplored. In the present study, exogenous ABA treatment obviously promoted fruit ripening as well as ethylene emission, whereas NDGA (Nordihydroguaiaretic acid, an inhibitor of ABA biosynthesis) application showed the opposite biological effects. Combined RNA-seq with time-course RT-PCR analysis, our study not only helped to illustrate how ABA regulated itself at the transcription level, but also revealed that ABA can facilitate ethylene production and response probably by regulating some crucial genes such as *LeACS4*, *LeACO1*, *GR* and *LeETR6*. In addition, investigation on the fruits treated with 1-MCP immediately after ABA exposure revealed that ethylene might be essential for the induction of ABA biosynthesis and signaling at the onset of fruit ripening. Furthermore, some specific transcription factors (TFs) known as regulators of ethylene synthesis and sensibility (e.g. *MADS-RIN*, *TAGL1*, *CNR* and *NOR*) were also observed to be ABA responsive, which implied that ABA influenced ethylene action possibly through the regulation of these *TF*s expression. Our comprehensive physiological and molecular-level analysis shed light on the mechanism of cross-talk between ABA and ethylene during the process of tomato fruit ripening.

## Introduction

Although the plant hormone abscisic acid (ABA) has been mainly regarded to function in non-climacteric fruit ripening [1], mounting studies have currently confirmed its regulatory role in climacteric fruit ripening [2–5]. A number of prior studies have indicated that exogenous ABA was able to accelerate the ripening process of some climacteric fruits (e.g. tomato, banana, peach, mango and melon) through pleiotropic biological effects on multiple ripening-related events [2–6]. For instance, ABA has been reported to directly participate in the cell wall catabolism to promote the softening of mango and tomato, which was via the regulation of a series of relevant enzymes and genes expression [5,7]. Besides, the application of exogenous ABA could facilitate tomato fruit coloring by enhancing carotenoids biosynthesis as well as chlorophyll degradation [2,8,9]. Previously, we have conducted a detailed morphological description and transcriptomic analysis about the possible effects of ABA on a suit of secondary metabolisms associated with tomato fruit ripening [10].

Accumulating evidence supports the idea that fruit ripening is not simply modulated by individual hormone, but is under the regulation by a complicated network of feedback and crosstalk among different phytohormones [11,12]. It has been well accepted ethylene and ABA play fairly important roles in control of fruit ripening [11,13]. Numerous studies have observed that ABA accumulates preceding ethylene release in climacteric fruits, implying ABA may function as an upstream regulator of ethylene biosynthesis and response [2,3,14,15]. ABA was found to exert antagonistic influence on ethylene synthesis and sensibility before the endogenous ABA reaches its peak level at the immature stage, but the inhibited effect would gradually weaken as the ABA increased [16,17]. When endogenous ABA elevated to a certain level, it could even promote the transformation of ACC (the full names for all abbreviations presented in the article can be referred in S10 Table, similarly hereinafter.) to ethylene [18–20]. It has been reported that ABA may act as an original trigger for the initiation of fruit ripening by inducing ethylene-mediated pathway and other ethylene-independent processes [2,3,5,21]. However, recent studies about the cross-talk between ABA and ethylene have been extensively demonstrated through measuring the hormone contents and activities of relevant enzymes after exogenous ABA/NDGA treatments [2,3,5,9,21]. There is currently no integrated analysis covering elements involved in the biosynthesis and signaling of these two hormones at the molecular level.

As a typical climacteric plant, tomato has been proven to be an excellent model system for analyzing the role of phytohormones in modulating fruit ripening and development. With the availability of tomato genome [22], it has been accessible to detect all expressed genes at the genome-wide level under various conditions. In recent years, next generation sequencing has been widely adopted to investigate the regulatory mechanism of multiple hormones by comparative transcriptome analysis. Zouine et al. have conducted a transcriptome profiling of tomato fruit to characterize several *ARF* genes which were regulated by both ethylene and auxin, implying the contribution of *ARF*s to the cross-talk between these two phytohormones [23]. Relying on RNA-seq technology, many genes involved in ABA and ethylene response were observed differentially expressed in mycorrhizal tomato fruits, elucidating the role of hormone networks in tomato mycorrhization [24]. Besides, a comparative sequencing analysis has been performed on tomato leaves, which screened the candidate genes related to ethylene signaling in response to exogenous ABA [25]. Currently, however, there is a lack of transcriptomic information available for the interaction between ABA and ethylene in regulating ripening process of tomato fruits.

In the present study, we planned to explore how ABA affects ethylene production and response through altering the endogenous ABA level by treatment with exogenous ABA and NDGA. Although there exist many ABA-deficient mutants (i.g. *notabilis*, *hp3*, *sitiens* and *flacca* etc.), the lower level of ABA would generally lead to abnormal growth and development of these mutant tomatoes [16,26–29]. Beside, treatment of ABA-deficient mutant with exogenous ABA could not alleviate the hormone deficiency phenotype [27]. Given the reduced size and weight of ABA-deficient mutants which may influence experiment results, we preferred to repress the endogenous ABA by NDGA which has high permeating speed and good efficiency in inhibition of ABA accumulation. We studied the responses of typical biochemical and physiological processes in ripening tomato to the alteration of ABA levels. Since the fruits sampled at the 9^th^ day after treatment represented a well-characterized stage of breaker, we used RNA-seq to conduct a transcriptomic profiling of all components involved in biosynthesis and signaling of these two hormones in different treatment samples at this stage, and identified the genes in response to ABA which were further verified with a ripening time-course analysis by RT-PCR. In addition, we also explored how ethylene affected ABA action at the onset of ripening by treating the fruits with 1-MCP immediately after ABA application. Furthermore, we analyzed the ripening-related TFs from RNA-seq data to explore possible involvement of TFs in the interplay between ABA and ethylene.

## Materials and Methods

### Plant material and treatments

Cherry tomatoes (*Lycopersicon esculentum var*. *cerasiforme* ‘XinTaiyang’) were grown in the greenhouses of Transfar Agriculture Co., Ltd (Xiaoshan, Zhejiang, China), which provided a standard culture temperature from 20°C to 25°C and relative humidity (RH) from 70% to 85%. With the permission of the company manager Li Laichun, fruits at mature green (MG) stage were harvested in June 2014 and immediately transported to the laboratory under ambient conditions. For sampling, fruit sepal and seeds were discarded and the dissected pericarps were quickly frozen in liquid nitrogen and stored at -80°C before subsequent analysis.

### Effect of exogenous ABA and NDGA treatments on fruit ripening

**Fig 1:**
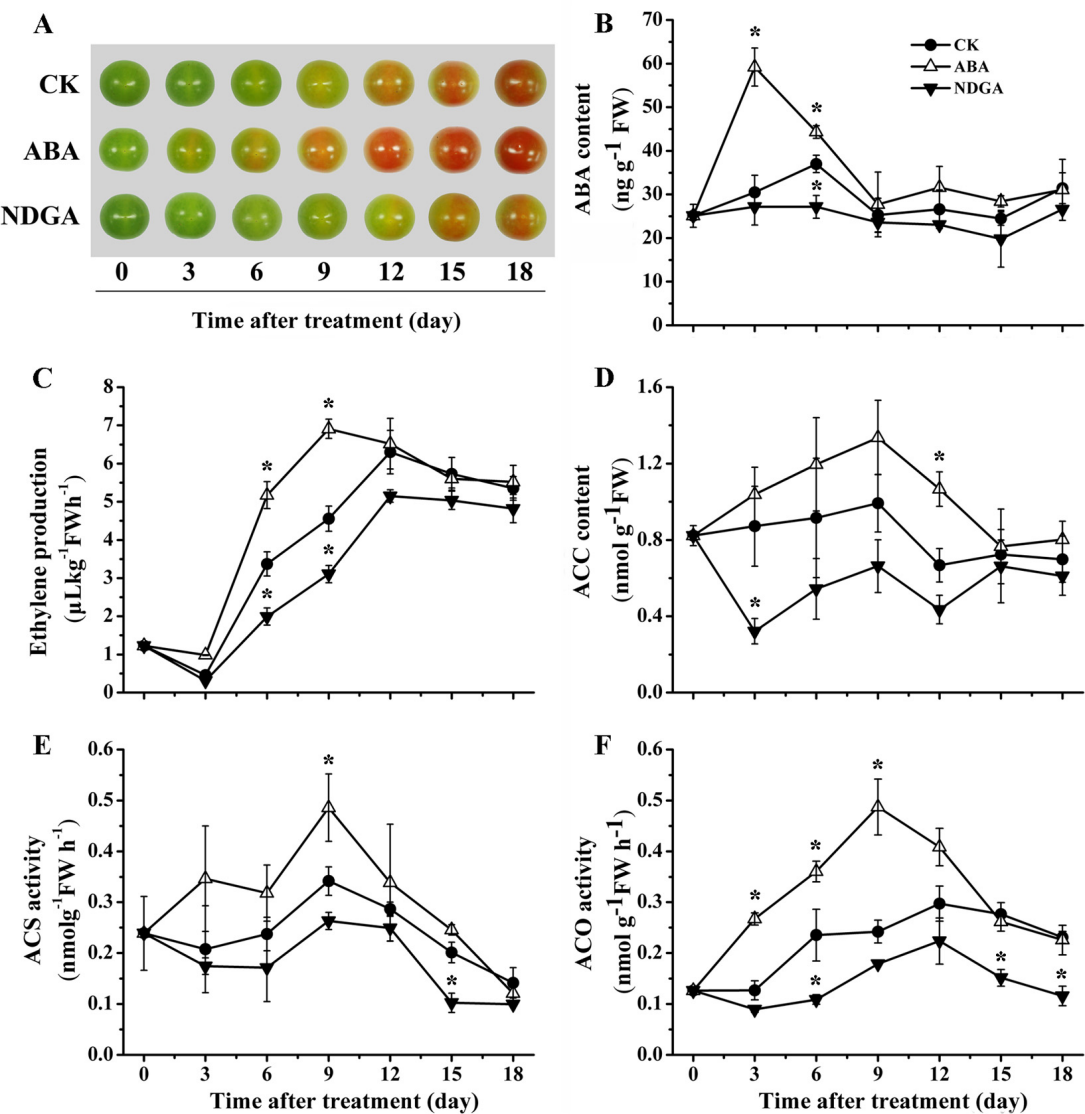
Effects of exogenous ABA and NDGA treatments on tomato phenotypes and phytohormone-related physiological indexes during storage at 20°C. (A) The morphological differences between the tomato fruits treated with exogenous ABA or NDGA and the non-treated fruits (CK). (B) Changes in ABA content as influenced by exogenous ABA and NDGA treatments during tomato ripening. (C) Changes in ethylene production as influenced by exogenous ABA and NDGA treatments during tomato ripening. (D) Effects of exogenous treatments on ACC content in tomato fruits. (E) Effects of exogenous treatments on ACS enzyme activities. (F) Effects of exogenous treatments on ACO enzyme activities. Vertical bars represents SE of three biological replicates, and asterisks (*) indicates significant difference (P < 0.05) between the value in ABA or NDGA treated fruits and that in control (CK).

The MG fruits of uniform size and free from external blemishes or infections were selected and divided into three groups in random. With the utilization of sterilized micro-syringe, the fruits of each group were uniformly injected with 25 μL aqueous solution of either ABA (10 mM) or NDGA (1 mM), and distilled water was served as the control. The injection method and the ABA/NDGA concentrations were applied as the optimum, which were obtained on the basis of a sum of preliminary experiments. After treatments, fruits were then stored at 20°C, 90% RH in the dark for 18 days. Fruits of each treatment were sampled every 3 days for measurement of ABA content, ethylene production, ACC content and ethylene biosynthesis enzymes. During the storage, samples on the 9^th^ day after treatments, corresponding approximately the breaker stage of tomato fruit ripening, were selected as the well characterized stage for RNA-sequencing, which presented the most evident distinctions in ripening process among the three treatments (Fig 1).

### Effect of exogenous ABA and ABA+1-MCP treatments on fruit ripening

We also set another three groups of fruits to further explore the interplay mechanism between ABA and ethylene in ripening process. By using the injection method described above, the tomatoes of two groups were treated with 25 μL per fruit each of exogenous ABA (10 mM), and the third group of tomatoes was injected with the same amount of distilled water in control. Then one of the group injected with exogenous ABA was treated immediately with 2 μL L^-1^ 1-MCP for 8 h (preliminary tests have showed the concentration and treatment time of 1-MCP was an ideal method to block ethylene response). The required volume of 1-MCP gas was generated by adding water to powdered formulation in a 15 L desiccators with fruits sealed inside. To reduce experimental errors, the fruits of other two groups (CK and ABA) were also sealed in the same volume of airtight desiccators for the desired exposure period (8 h) respectively, but were not exposed to 1-MCP. Following the treatments, all the fruits were removed from the glassy container and then stored at 20°C, 90% RH. Samples of the three groups were collected every 3 days for the determination of the content of ABA and ethylene production.

### Ethylene production

The rates of ethylene produced by whole fruit during ripening were determined according to Bu et al. with slight alterations [30]. Ten cherry tomatoes were enclosed in a 2.0 L airtight container for 2 h at 20°C. A headspace gas sample (1.5 milliliter) was withdrawn using a syringe, and then injected into a gas chromatography (SHIMADZU, GC-2014C PF, Japan) equipped with a flame ionization detector (FID), and a 2000×3 mm column of aluminum oxide at 85°C. Ethylene production was presented as μL∙Kg FW^-1^∙h^-1^, and the measurement was conducted in three replicates.

### Determination of ACC content

ACC content was measured according to Zhang et al. with minor modifications. Powdered pericarp samples (1 g) were homogenized on ice in 10 ml 95% aqueous ethanol (v/v), and then centrifuged at 12000×g for 15 min at 4°C [2]. The supernatant was evaporated to dryness under vacuum at 40°C and the residue was dissolved in 5 ml distilled water. After oxidative conversion, ACC content was determined through ethylene production by gas chromatography (SHIMADZU, GC-2014C PF, Japan) as Lizada and Yang described [31]. ACC concentration of each sample was expressed in nmol∙g^-1^ FW and the analysis was repeated three times.

### Determination of ACS activity

The determination of ACS activity was based on the method of Li et al. [32]. Pericarp sample (1 g) was added into 5 ml 0.1 mol L^-1^ phosphate buffer (pH = 8.0) containing 1 mmol L^-1^ EDTA, 1 mmol L^-1^ PMSF, 4 mmol L^-1^ DTT, 3% PVPP and 10μmol L^-1^ pyridoxal phosphate. The extracts were then centrifuged at 12000 × g for 15 min at 4°C. Then 0.5 ml supernatants were added into 1.5 ml HEPES-KOH buffer (pH = 8.0) which contained 250 μmolL^-1^ SAM and 10 μmolL^-1^ pyridoxal phosphate for further enzyme assays. The ACS activity was presented as nmol∙g^-1^ FW∙h^-1^, and the determination was conducted three times.

### Determination of ACO activity

The method described by Zhang et al. was adopted to measure ACO activity [2]. Fruit pericarp sample (1g) was homogenized in 5 ml solution containing 0.1 M Tris-HCl pH 7.5, 10% glycerine, 30 mM sodium ascorbic acid, 5% polyvinylpyrrolidone (PVP), 5 mM dithiothreitol (DTT), 0.2 mM FeSO_4_, and centrifuged at 12000 × g for 15 min at 4°C. Then 0.5 ml above solution was added into 1.5 ml enzyme solution which contained 10% glycerine, 30 mM sodium ascorbic acid, 2.0 mM ACC, 0.1 mM FeSO_4_ and 30 mM NaHCO_3_, kept at 30°C for half an hour. 1.5 mL headspace gas was injected into the gas chromatography for ACO activity measurement, and the unit was nmol∙g^-1^ FW∙h^-1^. The assay was conducted triplicates.

### Determination of ABA content

ABA was extracted from 1.5 g fruit pericarp tissue using 20 ml 80% methanol (v/v) at 4°C for 12 h, mixed with 1% polyvinylpyrrolidone (PVPP, w/v) as an antioxidant. After centrifugation (12000 × g, 15 min), the supernatant liquid was evaporated under reduced pressure at 35°C. The residue was dissolved in 9 ml 10% methanol − 0.4% acetic acid, and was further eluted through a Sep-Pak C18 cartridge (Waters, Milford, MA, USA) to remove polar compounds. Then the collected solution containing free ABA was also evaporated under vacuum at 35°C. The resulting dried precipitate was dissolved in 1.5 ml of 50% methanol, finally filtered through a 0.45 μm filter membrane and submitted to high-performance liquid chromatography (HPLC, Shimadzu, Kyoto, Japan) for ABA analysis. The mobile phase consisted of 0.4% (v/v) acetic acid (solvent A) and 100% methanol (solvent B). It was eluted with a linear gradient of methanol (45%–60%) at a flow of 1 mL∙min^-1^, and the detection wavelength of ABA was 260 nm. The external calibration curves were used for quantification.

### RNA extraction, Library construction and Illumina sequencing

Two batches of tomatoes were harvested from two successive weeks in June 2014, which were independently subjected to exogenous treatments (ABA, NDGA and CK) respectively to represent as two biological replicates for sequencing. For each treatment, equal quantities of high-quality RNA which was extracted from 10 individual fruits in the same batch respectively were pooled to form one biological replicate, and so there were two biological replicates for RNA-seq analysis. Total RNA was extracted with the utilization of Total RNA Purification Kit, TRK1001 (LC Science, Houston, TX) following the manufacture’s protocol. Then the quantity and purity of total RNA were evaluated by Agilent 2100 Bioanalyzer and RNA 6000 Nano LabChip Kit (Agilent, CA, USA), and only the sample with RIN (RNA integrity number) ≥ 8.5 was identified as qualified. Magnetic beads with oligo (dT) were used to isolate poly (A) messenger RNA, and divalent cations were added to cut the mRNA into short fragments under elevated temperature. Taking the cleaved RNA fragments as templates, first- and second-strand cDNAs were synthesized using mRNA-Seq sample preparation kit (Illumina, San Diego, USA), and the average length for the final cDNA libraries was 300 bp (±50 bp). After that, the paired-ended sequencing was performed on the six samples (two biological replicates) by Illumina Hiseq2000platform at the LC-BIO (Hangzhou, China) following the vendor’s instruction, generating 374.73 million reads and totally 37.43 Gbps. All the raw sequence data has been deposited at NCBI Sequence Read Archive (SRA) with the accession number GSE75276.

### Bioinformatics analysis

Raw reads obtained by Hiseq2000 were filtered to remove low quality reads (i.e. reads containing sequencing adapters; reads containing Ns (unknown sequence) > 5; nucleotide with q quality score lower than 20). The resulting clean reads were submitted for mapping analysis against the tomato genome (ftp://ftp.jgi-psf.org/pub/compgen/phytozome/v9.0/Slycopersicum/asse-mbly/) by using Tophat package [33]. The aligned reads were then processed by Cufflinks version 2.1.1, which were used for further differential expression analysis [33]. The relative abundance of genes was normalized with values for fragments per kilobase of exon per million fragments mapped (FPKM), and the genes expression level in each treatment was all expressed as the average of the two biological replicates. The pairwise comparing mRNA abundance of genes was performed in CK vs ABA and CK vs NDGA treatment, respectively. The genes were identified to be significantly differentially-expressed when their │log_2_fold change│≥1 and P value <0.05, while the genes differed by less than 20% (│log_2_ fold change│< 0.25) were deemed to not change in transcriptional level, and the other genes were assumed as slightly changed [25]. Besides, using the heatmap command in Multi Experiment Viewer 4.8 (MEV 4.8), the relative gene expression also can be analyzed by Z-score, which was determined according to the formula: *Z* = (*X*-*μ*)/σ, where *X* is the Log2-transformed relative FPKM value of a gene in a specific sample, and *μ* and *σ* represent the mean expression level and standard deviation (SD) of a gene across all samples, respectively [34,35].

### Quantitative Real-Time PCR (qRT-PCR)

The qRT-PCR was used not only to measure the transcriptional abundance of genes involved in hormone biosynthesis and signaling across the time course, but also to verify the expression patterns of the 9^th^ day fruits revealed by the RNA-seq analysis. For each treatment at a specific time point, the total RNA was extracted from 10 randomly selected fruits and was subsequently blended at equal quantity. Then, the three pools of RNA (ABA, NDGA and CK), one representing each treatment at a specific time point, were reverse-transcribed to cDNA with RNA PCR kit (TaKaRa, Japan), respectively. The qRT-PCR was performed on ABI StepOne RT-PCR system in which cDNA was used in 10μL reaction with SYBR Premix ExTaq (TaKaRa, Dalian, China) following the manufacture’s procedure. The relative expressions of genes were calculated with 2^-ΔΔCT^ method by normalizing to the internal control gene β-actin (Accession NO.U60481.1) [36]. Three replicates were conducted in qRT-PCR analysis, and primer sequences for the analyzed genes were presented in S9 Table. With respect to the validation of transcriptiome data, Pearson’s correlation test was adopted to analyze the correlation significance of genes expression in ABA and NDGA-treated fruits relative to the CK between qRT-PCR and RNA-seq.

### Correlation network analysis

A correlation network analysis of ABA and ethylene was conducted based on expression levels of the crucial genes and the physiochemical data, which were measured in different treatments at the key time points during ripening of Day 6, 9, 12 and 15. The present network was determined by the following parameters: the value of Pearson correlation coefficient (ρ), node strength (ns) and the network strength (NS) [37]. As the lines joining the nodes represented correlations, the positive correlations (ρ > 0) were shown in red, while the negative correlations (ρ < 0) were in blue. The edge thickness indicated the strength of the correlation, which was proportional to |ρ| of that particular pair [37]. The node size was proportional to ns which was the average of the |ρ| of a given node with all other nodes (ns = Avg |ρ|), and the NS was the mean of all node strengths (NS = Avg |ns|). The network diagram was visualized as “organic layout” by Cytoscape version 2.8.3 (www.cytoscape.org) [38].

### Statistical analysis

For all the biochemical determinations, the statistical analysis were performed with SPSS software and the values were expressed as mean ± standard error (the error bars in figures represented SE of three biological replicates). The significant differences between the ABA or NDGA treatment and CK by the least significant difference test (LSD) for P< 0.05 were indicated with asterisks.

## Results

### Effects of exogenous ABA/NDGA treatments on tomato ripening and fruit sampling for RNA-seq

Generally, significant physiological and morphological distinctions were observed among the tomatoes with different treatments during the 18 days’ storage (Fig 1). In comparison with control fruits, the application of ABA obviously accelerated ripening process including a series of relevant biological events which have been discussed in our previous study [10], whereas exogenous NDGA treatment led to a observable postponement of ripening in tomato fruits (Fig 1A). As expected, the ABA accumulation in ABA-treated fruits was considerably increased and reached the maximum level 3 days earlier than in control fruits (Fig 1B). In contrast, exogenous NDGA could significantly block the ABA biosynthesis, which consistently suppressed ABA content to a quite low level (Fig 1B). On the other hand, the ethylene production was substantially induced after ABA treatment and reached the peak at the 9^th^ day, which was precedent relative to that in CK fruits (Fig 1C). A noticeable reduction of ethylene was detected in NDGA-treated fruits, but the inhibition by NDGA was alleviated in the later ripening stage because of the limited period of time for NDGA effectiveness (Fig 1C). As precursor of ethylene, the alterations of ACC content showed a similar trend as ethylene production in response to ABA/NDGA treatments (Fig 1D), consistent with the changes of ACS and ACO activities affected by exogenous applications (Fig 1E and 1F). Based on the results described above, we therefore selected the fruits sampled at the 9^th^ day, which on average corresponded to around beaker stage of tomato fruit (Fig 1A), as the key time point for RNA-seq analysis, to further investigate the molecular mechanism underlying the influence of ABA on its biosynthetic and signaling pathway as well as that of ethylene during fruit ripening.

### A snapshot of tomato mRNA profiling with exogenous treatments

To conduct comparative transcriptome analysis of tomato in response to exogenous applications of ABA and NDGA during fruit ripening, we adopted RNA-seq to generate expression profiles for each of the three groups (ABA, NDGA and CK). Two independent biological replicates were collected for each group, and the principle component analysis (PCA) revealed relatively low biological variability within individual replicates (S1A Fig). After filtering low quality reads, the clean reads ranging from 46.07 to 79.43 million for each cDNA library were mapped to the tomato genome sequence SL2.40 (ITAG2.3) with Tophat (S1 Table) [39]. As shown in S1 Table, 43.21–60.05 million reads were aligned to the reference sequence, accounting for 93.17% of the total reads. Besides, an average of 89.98% of CK, 90.10% of ABA and 89.92% of NDGA reads were matched to a unique genomic location in tomato genome (S1 Table). In our data, a total of 31571 genes expressed in the tomato fruit transcriptome, including 28399 genes in CK, 28161 in ABA and 29875 in NDGA, respectively (S2 Table). In the comparison between CK and ABA-treated samples, the expression of 1256 genes were found to be significantly changed (│log2 fold change│ ≥ 1 and P value < 0.05), including 358 up-regulated and 898 down-regulated genes (S3 Table, S1B Fig). After the treatment with exogenous NDGA, 1091 genes were significantly altered in transcription level compared with control fruits, including 818 up-regulated and 273 down-regulated genes (S4 Table, S1B Fig). With regard to the DEGs, there were more down-regulated genes than up-regulated ones in ABA treatment and the reverse were found in NDGA treatment (S1 Fig), which implied that many genes may respond negatively to ABA in tomato fruit.

To obtain insights into the transcriptional regulatory mechanism of ABA in depth, we identified 1343 transcription factor (TF) genes from 57 families (S5 Table, S2A Fig) according to the PlantTFDB database (http://planttfdb.cbi.pku.edu.cn) by BLAST [40]. Among the detected TFs, a total of 122 genes were differentially expressed in response to exogenous treatments (72 DEGs in ABA and 53 DEGs in NDGA treatment) (S5 Table). Most TFs were down-regulated in ABA-treated fruits and induced by NDGA application, including the genes with well-known involvement in fruit ripening, such as AP2, C2H2, ERF, G2-like, HD-ZIP, MIKC, MYB-related and SBP families (S2A and S2B Fig) [41,42].

### The overall expression profiles of ABA metabolism and signaling genes

**Fig 2:**
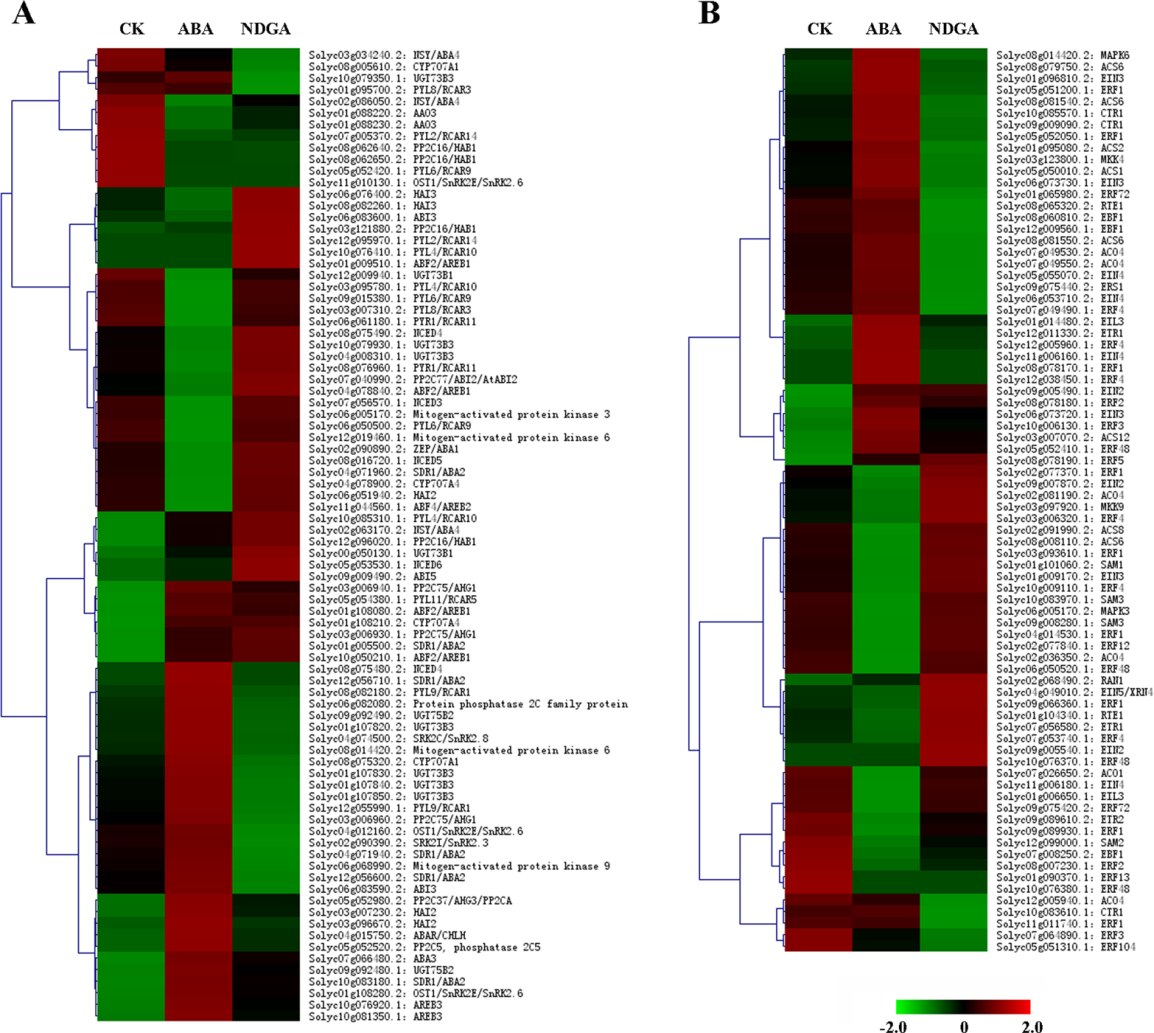
The overall transcriptional profiles of genes involved in biosynthesis and signaling of ABA and ethylene, respectively. (A) The Fig showed an overview of genes expression related to ABA biosynthesis, deactivation and signaling in different treatments. (B) The Fig showed an overview of genes expression related to ethylene biosynthesis and signaling in different treatments. Analysis of the relative gene expression value (Z-score) was performed using the heatmap command in MEV 4.9.0. Red and green colors indicate relative higher and lower expression values, respectively.

**Fig 3:**
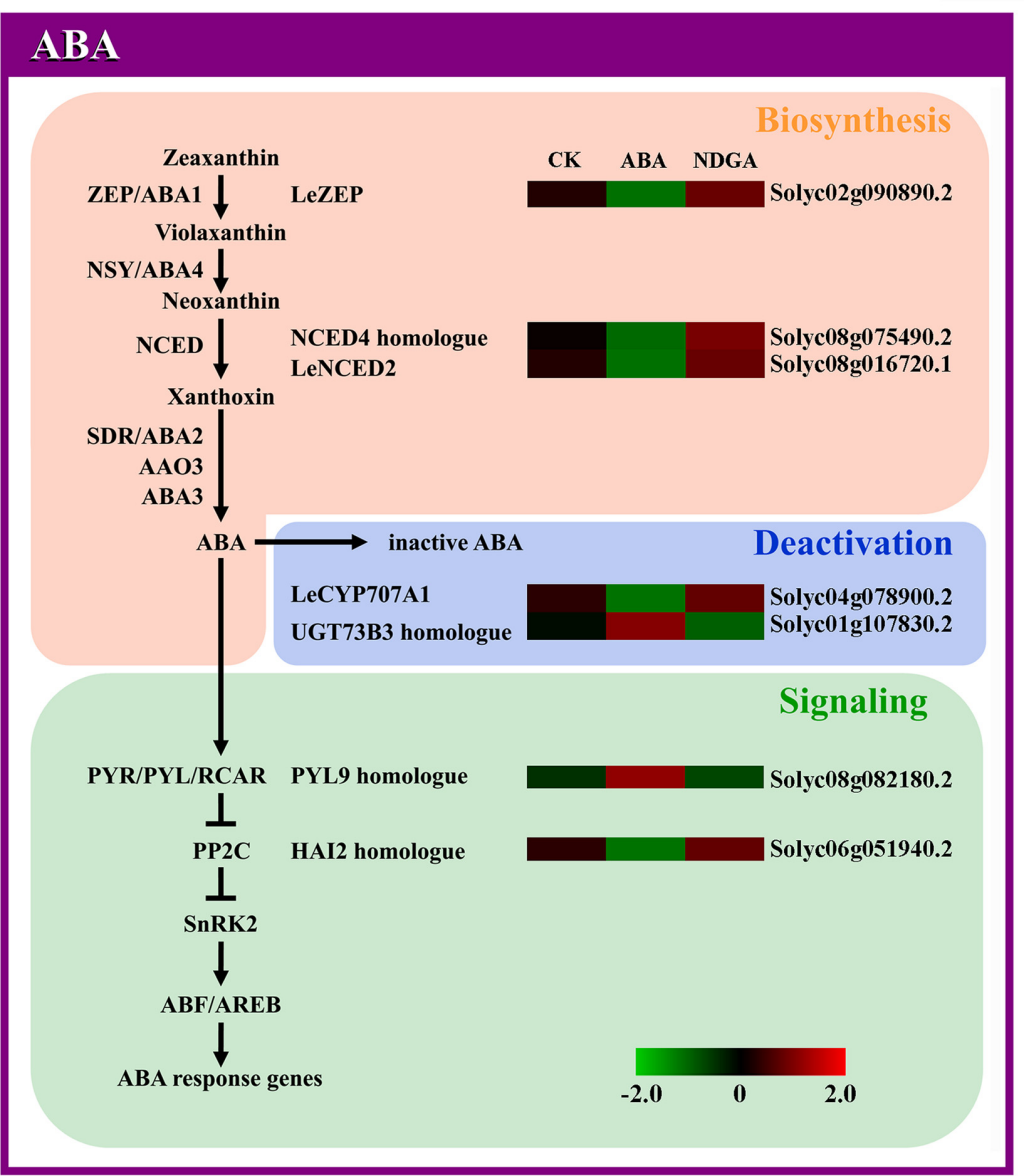
Analysis of DEGs involved in the pathway of ABA biosynthesis, deactivation and signaling. Abbreviations are listed in S10 Table, and other details are the same as in Fig 2.

From RNA-seq data, a total of 84 genes involved in ABA metabolism and signal transduction were identified (Fig 2A, S6 Table). In the ABA biosynthetic pathway (Fig 3), zeaxanthin was epoxidized to violaxanthin by ZEP in a two-step oxidation [43], and the synthesized violaxanthin is subsequently converted to neoxanthin with the catalysis of NSY [44]. NCED functions as a rate-limiting enzyme for ABA biosynthesis, which catalyzes oxidative cleavage of carotenoid to produce C15 intermediate xanthoxin [45]. Then with the reduction of SDR, xanthoxin is converted to ABA-aldehyde [46], which finally forms ABA after oxidation by AAO3 [47]. Besides, molybdenum cofactor (MoCo), which is encoded by *ABA3*, has been reported to be essential for the catalytic activity of AAO3 [48]. In total, there were 1 *ZEP*, 3 *NSY*s, 5 *NCED*s, 6 *SDR*s, 2 *AAO3*s and 1 *ABA3* identified in our transcriptome data (Fig 2A, S6 Table). As the first step of ABA biosynthesis [49], the transcription abundance of *LeZEP* was significantly down-regulated in ABA treatment, and slightly up-regulated from 27.79 to 43.00 FPKM in NDGA treatment (Fig 3, S6 Table). Although *LeNCED1* has been reported as the key gene involved in ABA biosynthesis [50], our data have detected other two *NCED*s showing significantly different expression levels in response to exogenous ABA/NDGA treatments. The *NCED4* homologue (Solyc08g075490.2) was observed more highly expressed in NDGA-treated fruits (from 0.69 to 2.39 FPKM) accompanied by slight repression in ABA treatment (Fig 3, S6 Table). Similarly, an obviously lower level of *LeNCED2* (Solyc08g016720.1) transcripts was detected after ABA application, while the expression was slightly induced 1.46 fold in the fruits treated with NDGA (Fig 3, S6 Table). As for the other genes encoding key elements in ABA biosynthesis, there was not a great deal of difference in transcription with external applications of ABA/NDGA (S6 Table).

The pathways of ABA catabolism are mainly categorized into two types, oxidation and conjugation (Fig 3) [51]. The predominant step of oxidative degradation is the hydroxylation at 8’ position of ABA to yield 8’-OH-ABA, which is mediated by CYP707A (ABA8OX) [52,53]. Even though *LeCYP707A2* has been accepted as the key gene in regulating ABA catabolism [50], the *LeCYP707A1* (Solyc04g078900.2) was identified as DEG in our data, which showed considerable down-regulation from 2.43 to 0.37 FPKM by ABA, and slight elevation by 1.76 fold in NDGA treatment (Fig 3, S6 Table). In addition, the formation of ABA conjugates (i.e. ABA-GE and ABA-GS) appears to be an alternative means for ABA deactivation [54], and the ABA glucosylation can be catalyzed by UGT [55]. In our data, the expression of *UGT73B3* homologue showed significant up-regulation with the application of ABA, and slight decline from 281.88 to 166.83 FPKM in NDGA treatment (Fig 3, S6 Table).

Recent studies have led to the construction of a core ABA signaling pathway (PYR/PYL/RCAR-PP2C-SnRK2) (Fig 3), which emerged as a double negative regulatory module [56]. In the absence of ABA, clade A PP2Cs physically interact with Subclass III SnRK2s, and efficiently repress the ABA signaling via dephosphorylation of SnRK2s [57]. However, the PP2C-dependent negative regulation are disrupted when ABA binds to PYR/PYL/RCAR receptors, allowing SnRK2s activation and subsequent phosphorylation of downstream targets (e.g ABF/AREBs) to trigger various ABA-induced physiological responses [58]. Among the 15 genes of ABA receptors (PYR/PYL/RCAR), one homologue of *PYL9/RCAR1* (Solyc08g082180.2) was identified as DEG, showing remarkably increased expression in ABA (2.16 fold) and little decreased level in NDGA-treated samples (Fig 3, S6 Table). Group A of PP2Cs have been reported as major negative regulators of ABA signaling [57], six members of which (i.e. ABI2, HAB1, AHG3, AHG1, HAI2 and HAI3) were identified in our sequencing data (S6 Table). Of the 14 *PP2C*s, one *HAI2* homologue (Solyc06g051940.2) was significantly down-regulated upon treatment with ABA, and slightly induced from 5.19 to 6.95 FPKM in NDGA treatment (Fig 3, S6 Table). With respect to the proteins belonging to Subclass III of SnRK2 family [59], only the genes encoding SRK2I (SnRK2.3) and SRK2E (OST1/SnRK2.6) were detected in tomato transcriptome (S6 Table). Although no significant difference was observed in *SnRK2*s expression with exogenous applications, most of them were slightly induced by ABA (from 1.14 to 1.34 fold) and repressed in NDGA treatment (from 1.26 to 1.70 fold) (S6 Table). In addition, the transcription of SnRK2 phosphorylation targets (e.g. *ABI5*, *AREB1/ABF2* and *AREB2/ABF4* etc.) did not seem to be affected much by external treatments either (S6 Table).

### The overall expression profiles of ethylene biosynthesis and signaling genes

**Fig 4:**
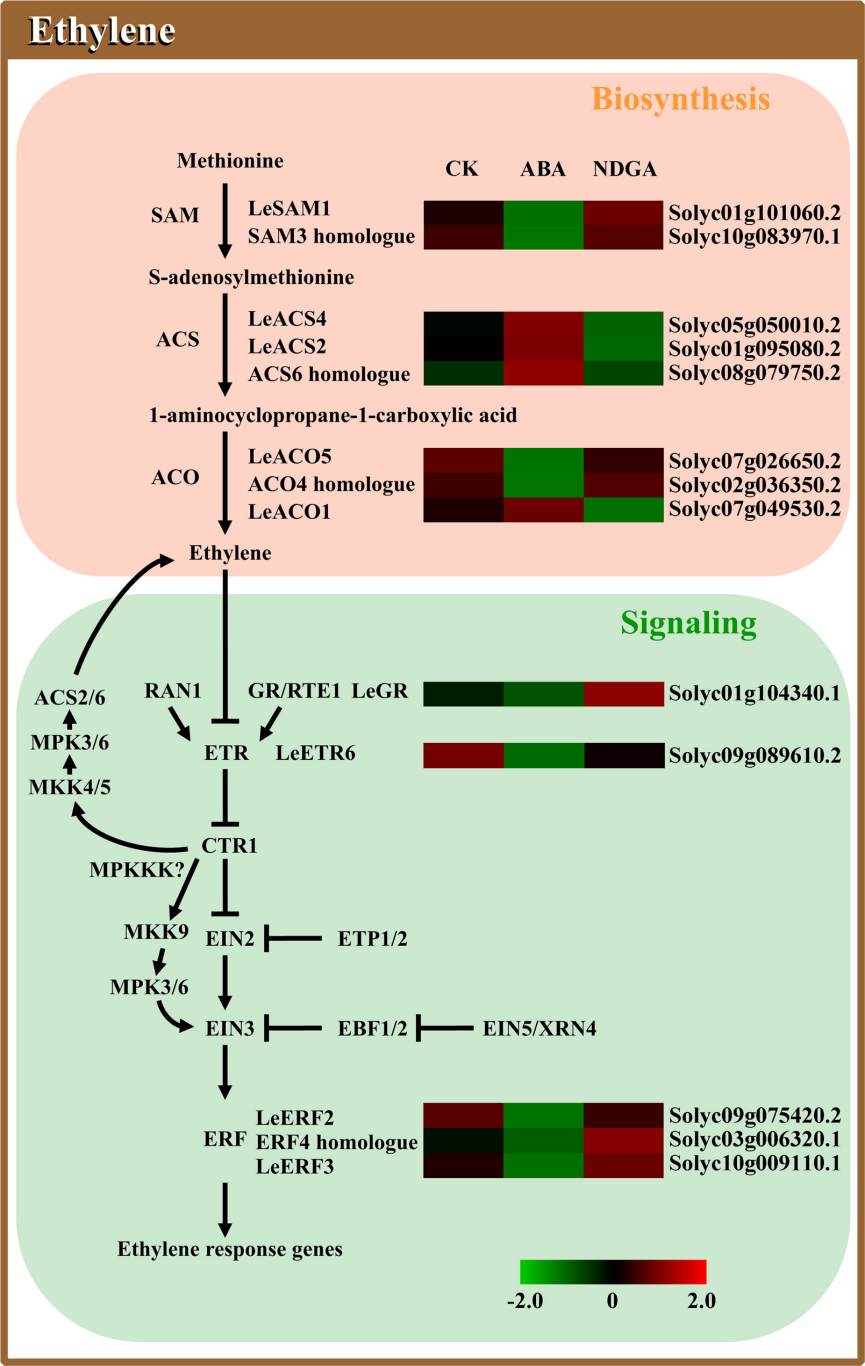
Analysis of DEGs involved in the pathway of ethylene biosynthesis and signaling. Abbreviations are listed in S10 Table, and other details are the same as in Fig 2.

A total of 78 genes related to ethylene biosynthesis and signal transduction were detected in our study (Fig 2B, S7 Table). Ethylene synthesis begins with the formation of SAM from methionine (Fig 4), with the catalyzation of SAMS [60]. By the action of ACS, SAM can be subsequently converted to ACC and finally form ethylene via the catalysis of ACO [61] (Fig 4). All the key enzymes of ethylene biosynthesis are encoded by multigene families, and 4, 9 and 6 genes are annotated with the function as SAMS, ACS and ACO, respectively (S7 Table). From sequencing data, 2 *SAMS*s (Solyc01g101060.2 and Solyc10g083970.1) were identified as DEG, both of which were evidently repressed by ABA, and slightly enhanced in NDGA-treated fruits (Fig 4, S7 Table). With regards to *ACS*s, the *LeACS4* (Solyc05g050010.2) showed remarkable up-regulation in ABA coupled with moderate reduction in NDGA treatment, and similar alteration was also observed in one analogue of *ACS6* (Solyc08g079750.2) (Fig 4, S7 Table). It has been reported that *LeACS2* (Solyc01g095080.2) encodes a crucial speed-restricting enzyme of ethylene synthesis in tomato fruit [62]. Our results showed that the expression of *LeACS2* was considerably induced 2.29 fold with ABA application and exhibited 2.6 fold reduction in NDGA treatment (Fig 4, S7 Table). Since the genes of *ACO* family have been reported to show different expression patterns in tomato ripening [63,64], a consistent phenomenon was also observed in our result (S7 Table). Of the six *ACO*s, the transcription of *LeACO1* (Solyc07g049530.2) in our data was coincide with numerous studies showing the highest abundance among all *ACO*s during tomato ripening [63,65], and the it was detected to be up-regulated from 2156.94 to 3828.27 FPKM in ABA and reduced approximate 4.0 fold at expression level upon NDGA application (Fig 4, S7 Table). However, the other two *ACO*s (Solyc07g026650.2 and Solyc02g036350.2) showed significant decrease in expression with exogenous ABA as well as slight increase with NDGA (Fig 4, S7 Table).

Considerable genetic and biochemical analyses have contributed to the generation of a primarily linear model for ethylene signaling, which starts from ethylene perception at the membrane and ends with transcriptional regulation in the nucleus (Fig 4) [66]. In the absence of ethylene, the ETR1 can be positively regulated by RTE1 to form receptor-CTR1 complex, which subsequently inhibits the interaction between receptors and EIN2 [67,68]. Of the two genes homologous to Arabidopsis *RTE1* identified in our data, one *RTE1* paralog (also called as *LeGr*, Solyc01g104340.1) was more abundantly expressed in NDGA treatment (from 2.05 to 9.54 FPKM), and slightly repressed 1.79 fold by ABA (Fig 4, S7 Table). In Arabidopsis, two F-box proteins ETP1/2 have been assumed to target EIN2 for degradation via 26S proteasome-dependent pathway [69]. However, there were no *ETP*s detected in tomato transcriptome (S2 Table). The activated CTR1 can directly phosphorylate the C terminal of EIN2, thus preventing signaling to downstream components [70]. Additionally, CTR1 also functions as a Raf-like MAPKKK and activates an unknown MAPK cascade to degrade EIN3 by phosphorylation, which finally suppresses ethylene response [71]. In the presence of ethylene, the receptors bind the hormone with the help of copper cofactor that is supplied by copper transporter RAN1 [72]. Then the receptor complex is disassociated concomitant with inactivated CTR1 releasing from ER membrane, which relieves the suppressive state of EIN2 [70]. The C end of EIN2 is cleaved off and translocates to nuclear where it stabilizes EIN3/EILs and initiates transcriptional response of ERFs [73]. In our study, eight genes homologous to ethylene receptors, and three each for *CTR1* and *EIN2* as well as six for *EIN3/EIL* were detected in tomato transcriptome (S7 Table). The *LeETR6* (Solyc09g089610.2) was found to be significantly down-regulated in ABA treatment (Fig 4), whose reductions in expression has been reported as critical induction for ethylene response [74]. However, no obvious changes at transcription level of *CTR1*s, *EIN2*s and *EIN3/EIL*s were observed in our study (S7 Table), indicating exogenous ABA or NDGA applications had inconspicuous effect on their expression. Among 41 expressed *ERF*s, *LeERF2* (Solyc09g075420.2), *LeERF3* (Solyc10g009110.1) and *LeERF4* (Solyc05g052030.1) were identified to be significantly down-regulated by ABA treatment, and one *ERF4* homologue (Solyc03g006320.1) was expressed 2.56 fold higher in NDGA treatment (Fig 4, S7 Table). Upon ethylene perception, dual MAPK cascades downstream of CTR1 have emerged as positive regulators in ethylene signaling [75]. The activation of MKK9-MPK3/6 module can promote EIN3 stabilization [71], and MKK4/5-MPK3/6 cascade leads to ACS2/6 accumulation which consequently enhances ethylene production [76]. The expression level of *MKK9* paralog (also called as *LeMKK4*, Solyc03g097920.1) was weakly suppressed from 10.86 to 7.00 FPKM by ABA and little induced to 19.69 FPKM with NDGA treatment (S7 Table). In contrast, *MKK4* homologue exhibited slight increase in expression by ABA and moderate reduction by NDGA (S7 Table). The downstream *MPK6* and *MPK3* homologues were found to be either slightly up- or down-regulated by ABA but both remain relatively constant in NDGA-treated fruits (S7 Table). Additionally, *LeEBF*s and *LeXRN4*, which encode post-translational regulators in ethylene signaling, did not differ in transcription abundance with exogenous ABA/NDGA treatments (S7 Table).

### RT-PCR analysis of representative genes related to ABA metabolism and signaling at different ripening stages

**Fig 5:**
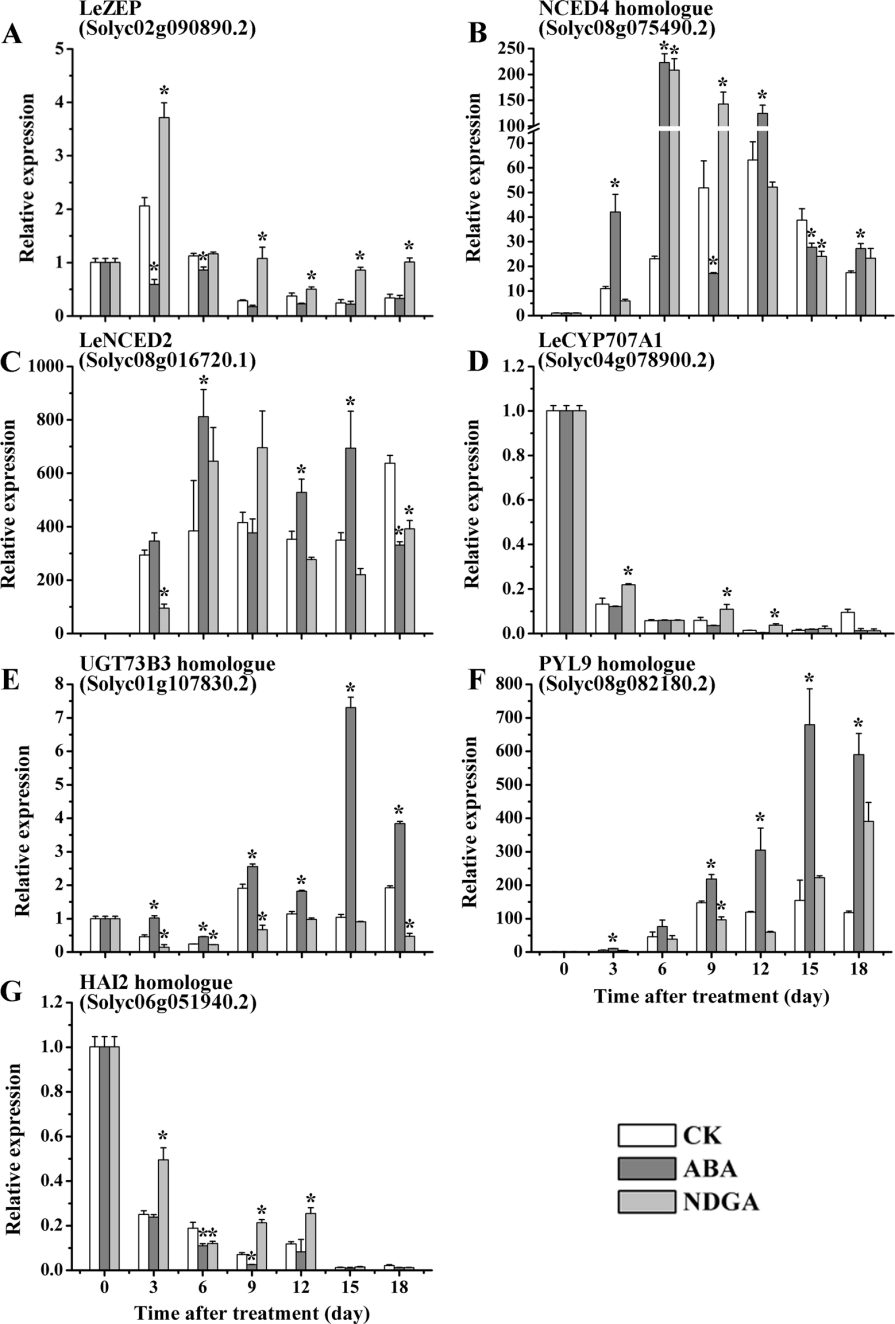
The expression of DEGs related to ABA metabolism and signaling in CK, ABA and NDGA-treated fruits at different ripening stages. RT-PCR analysis of *ZEP* (A), *NCED4* (B), *NCED5* (C), *ABA80X* (D), *UGT73B3* (E), *PYL9* (F) and *HAI2* (G) were conducted in CK vs ABA and CK vs NDGA treatment. Numbers under the *x-axis* represents the different time points during fruit ripening while *y-axis* shows relative expression in folds between CK and ABA/NDGA-treated fruits. Error bars represents SE of three biological replicates, and asterisks (*) indicates significant difference (P < 0.05) between the value in ABA or NDGA treated-fruits and that in control (CK).

From RNA-seq data of fruits sampled at the 9^th^ day, the identified DEGs were primarily regarded as representative genes for further analysis of dynamic expression patterns (S6 Table). In this study, three ABA biosynthetic genes and two deactivation related genes were selected to investigate transcription abundance at different time points of fruit ripening (Fig 5A–5E). Compared with CK, *LeZEP* (Solyc02g090890.2) was down-regulated by ABA treatment during ripening process, whereas the expression level in NDGA-treated fruits was significantly higher than that in control throughout all time points (Fig 5A). With respect to the *NCED4* homologue (Solyc08g075490.2) and *LeNCED2* (Solyc08g016720.1), the overall expression patterns were observed similar in response to different treatments (Fig 5B and 5C). In the early stage of ripening, the expression of *NCED4* homologue and *LeNCED2* were obviously elevated by exogenous ABA and reached a maximum level at the 6^th^ day (Fig 5B and 5C). Upon NDGA treatment, both of them were immediately suppressed at the 3^rd^ day, and subsequently showed a transitory increase between the 6^th^ day and 9^th^ day (Fig 5B and 5C). *LeCYP707A1* (Solyc04g078900.2) was involved in the oxidative degradation of ABA, whose transcription exhibited a continuously decreased trend as fruits ripened (Fig 5D). In comparison with CK, the gene in NDGA-treated fruit showed a delayed decline at expression level, while exogenous ABA accelerated the down-regulation of *LeCYP707A1* (Fig 5D). On the contrary, the expression of *UGT73B3* homologue, which functions in the formation of conjugated ABA, was considerably induced by ABA and repressed in NDGA treatment (Fig 5E). After application with exogenous ABA/NDGA, the homologues of *PYL9* (Solyc08g082180.2) and *HAI2* (Solyc06g051940.2) were detected to be differentially expressed in ABA signaling pathway (S6 Table). As an important receptor of ABA, up-regulation of *PYL9* paralog in ABA treatment was concomitant with down-regulation in NDGA-treated fruits throughout all ripening stages (Fig 5F). *HAI2* was known as a main negative regulator in ABA signaling, and its expression showed a reduction in ABA treatment and a significant increase in response to NDGA during ripening process (Fig 5G).

### RT-PCR analysis of representative genes related to ethylene biosynthesis and signaling at different ripening stages

**Fig 6:**
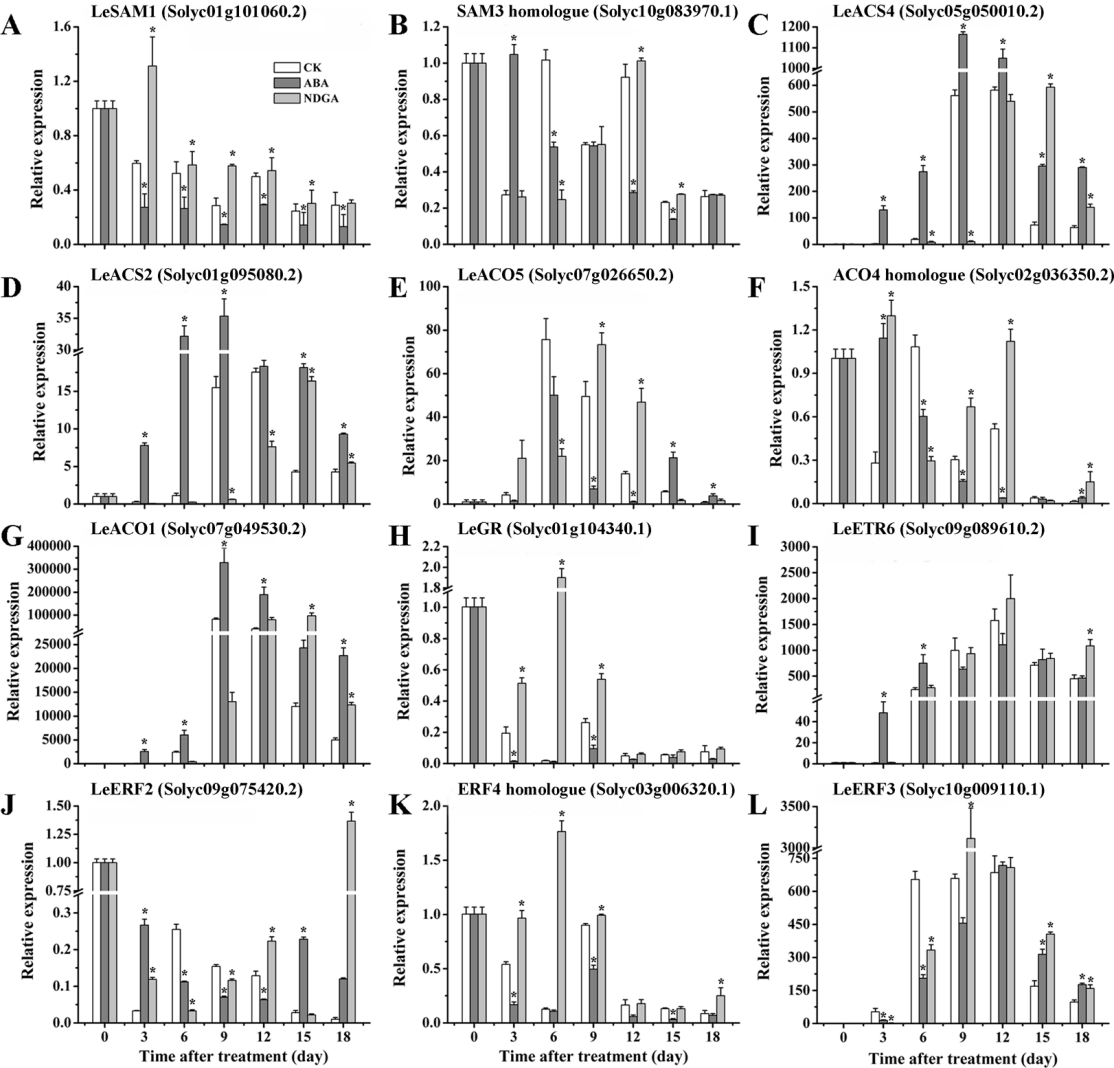
The expression of DEGs related to ethylene biosynthesis and signaling in CK, ABA and NDGA-treated fruits at different ripening stages. RT-PCR analysis of *SAM1* (A), *SAM3* (B), *ACS1* (C), *ACS2* (D), *ACO1* (E), *ACO4;1* (F), *ACO4;2* (G), *RTE1* (H), *ETR2* (I), *EBP* (J), *ERF4;1* (K) and *ERF4;2* (L) were conducted in CK vs ABA and CK vs NDGA treatment. Other details are the same as in Fig 5.

In the sequencing analysis, seven genes related to ethylene biosynthesis were found to be differentially expressed after exogenous ABA or NDGA treatments, including two *SAM*s, two *ACS*s and three *ACO*s (S7 Table). In contrast with CK, the expression of *LeSAM1* (Solyc01g101060.2) was considerably reduced by ABA and more abundant in NDGA-treated fruits at most developmental stages (Fig 6A). Distinct from *LeSAM1*, the transcript level of *SAM3* homologue (Solyc10g083970.1) showed a great increase with exogenous ABA and reached the peak at the 3^rd^ day, but it subsequently declined obviously through the remaining stages of ripening (Fig 6B). However, an obviously delayed expression was observed in NDGA treatment throughout fruit ripening (Fig 6B). From the RT-PCR data, the expression of *LeACS2* (Solyc01g095080.2) was detected with significant up-regulation by ABA and suppression by NDGA across the ripening time course (Fig 6C and 6D). Similar to *LeACS2*, the transcript abundance of *LeACS4* (Solyc05g050010.2) was also significantly enhanced by ABA treatment over the entire period of fruit ripening (Fig 6C). Nevertheless, the *LeACS4* transcription was considerably repressed by NDGA application until the 9^th^ day, and then began to increase at the later ripening stage (Fig 6C), which was probably in correlation with the increased ethylene production from Day 9 as the NDGA inhibitory effectiveness gradually eliminated (Fig 1C). Compared with CK, the peaks of these two *ACS*s transcription abundance were also advanced by about 3 days with the treatment of ABA. Among the three *ACO*s, *LeACO1* (Solyc07g049530.2) was expressed at the highest abundance, which was induced by ABA at all time points and continuously repressed by NDGA until the later stage of ripening (Fig 6G). Although fluctuated expression was observed in other two *ACO*s (*LeACO5* and the *ACO4* homologue) by exogenous treatments, both of their expression remained at quite low levels during tomato ripening (Fig 6E and 6F). Therefore, it can be supposed that *LeACO1* may play a predominant role among the three *ACO*s involved in the regulation of ethylene biosynthesis by ABA. With regard to ethylene signaling, five DEGs were selected for examination of expression level at various ripening stages (S7 Table). As a negative regulator in signal transduction, the expression of *LeGR* was remarkably inhibited by exogenous ABA and accumulated in a much higher amount with NDGA application (Fig 6H). At the early stage of ripening (Day 3–6), *LeETR6* (Solyc09g089610.2) was more highly expressed in ABA treatment, but at later stage (Day 12), the gene began to show increased transcription in NDGA-treated fruits (Fig 6I). Among all *LeERF*s analyzed in tomato fruits, *LeERF2* (Solyc09g075420.2) has been reported as the only one to exhibit ripening-associated expression [77,78]. The expression of the *LeERF2* was found to be highly expressed at the early stage by exogenous ABA and at the later stage by NDGA treatment (Fig 6J). Intriguingly, the expression of *ERF4* homologue (Solyc03g006320.1) was significantly down-regulated by ABA and induced by NDGA through all ripening stages (Fig 6K). While the *LeERF3* did not show consistent repression or activation by exogenous treatments, implying it may not be the crucial genes regulated by ABA in ethylene signaling pathway.

### Exploration of the interaction between ABA and ethylene in fruit ripening

**Fig 7:**
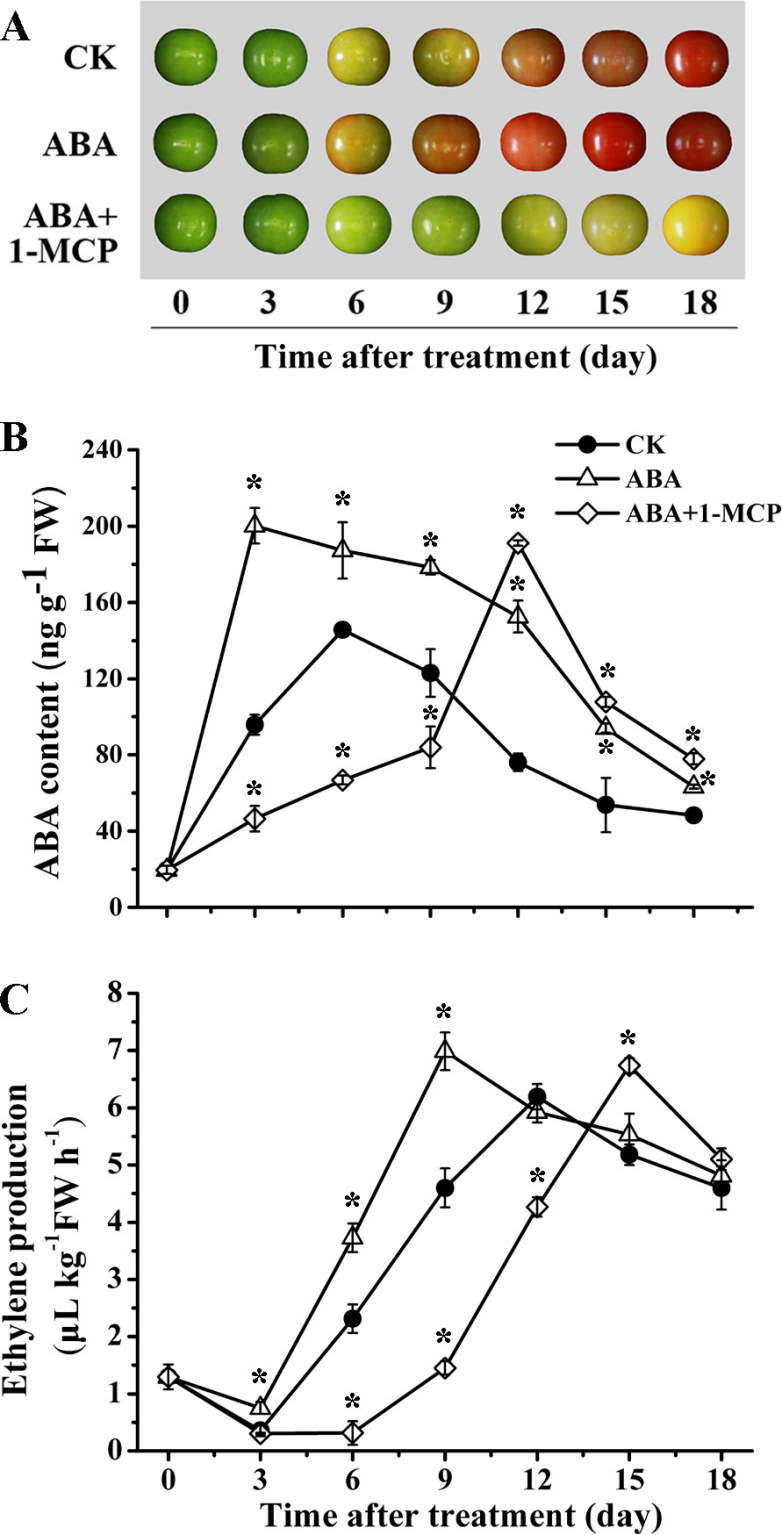
Effects of exogenous ABA and ABA+1-MCP treatments on tomato phenotypes and phytohormone contents during storage at 20°C. (A) The morphological differences between the tomato fruits treated with exogenous ABA or ABA+1-MCP and the non-treated fruits (CK). (B) Effects of exogenous ABA and ABA+1-MCP treatments on ABA content during tomato ripening. (C) Effects of exogenous ABA and ABA+1-MCP treatments on ethylene evolution during tomato ripening. Other details are the same as in Fig 1.

Contrary to the accelerated ripening found in ABA treatment alone, the fruits with ABA+1-MCP application were observed to be remarkably deferred in ripening progress (Fig 7A). The ABA accumulation in ABA+1-MCP treatment remained at a low level until day 9, and then increased rapidly to reach the peak on day 12, which was significantly delayed relative to that of CK and ABA-treated fruits, respectively (Fig 7B). However, there was no marked difference in the maximum level of ABA content between the fruits treated with ABA alone and together with 1-MCP, both of which were apparently higher than that in control fruits (Fig 7B). On the other hand, ethylene production in ABA+1-MCP group was almost completely blocked by 1-MCP for a quite long period (about 9 days after treatment), and then gradually increased to the peak level which was parallel with that in ABA-treated fruits (Fig 7C).

**Fig 8:**
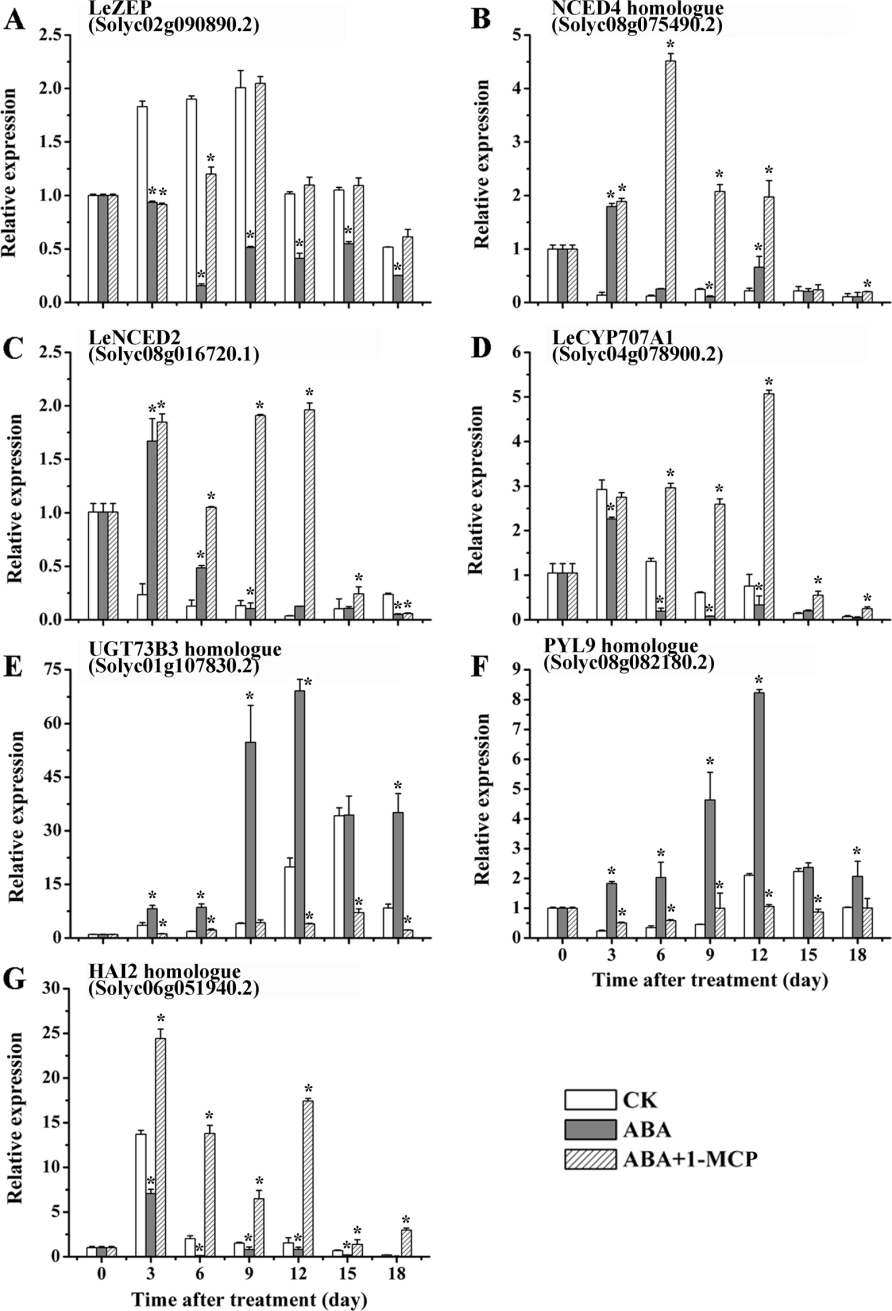
The expression of DEGs related to ABA metabolism and signaling in CK, ABA and (ABA+1-MCP)-treated fruits at different ripening stages. RT-PCR analysis of *ZEP* (A), *NCED4* (B), *NCED5* (C), *ABA80X* (D), *UGT73B3* (E), *PYL9* (F) and *HAI2* (G) were conducted in CK vs ABA and CK vs ABA+1-MCP treatment. Other details are the same as in Fig 5.

**Fig 9:**
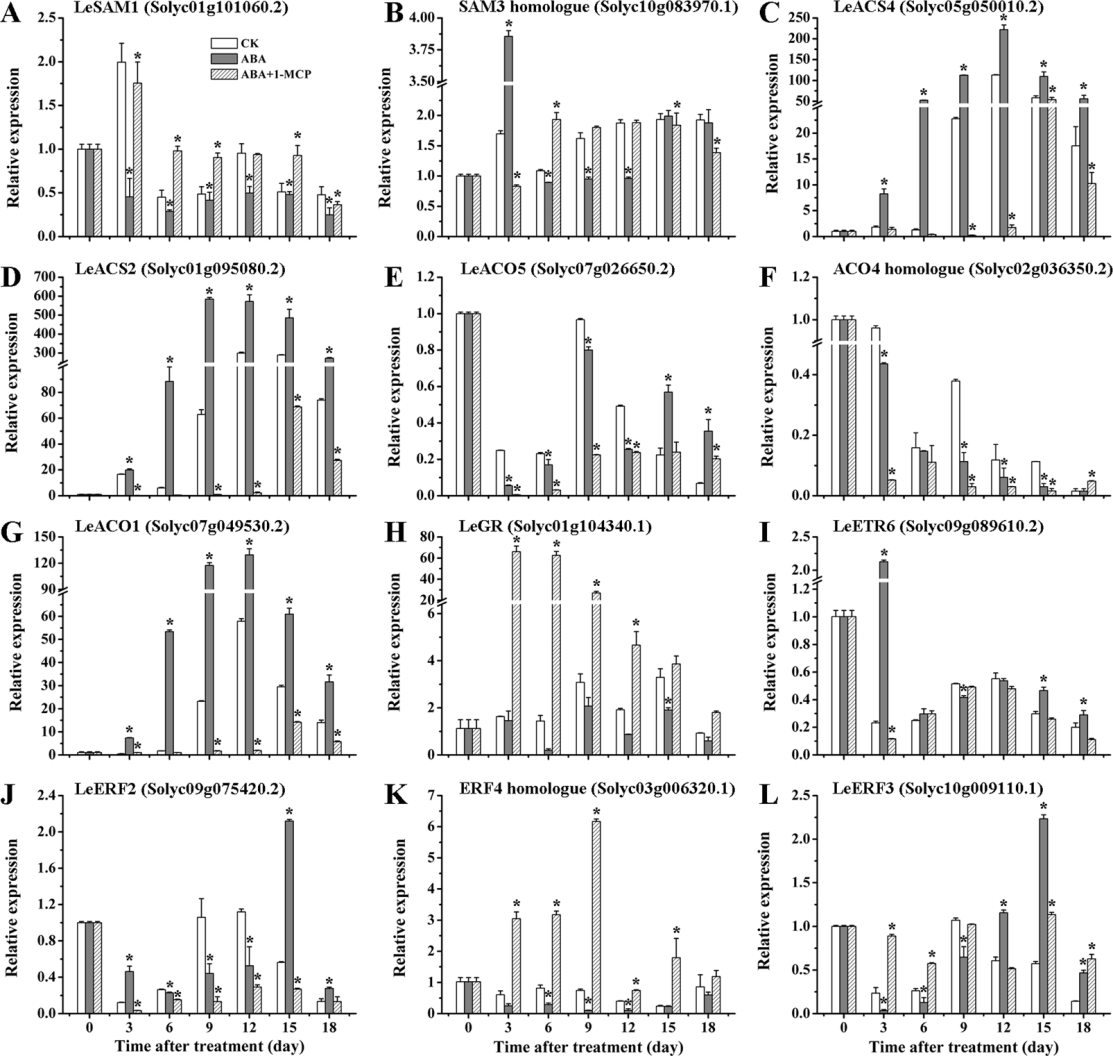
The expression of DEGs related to ethylene biosynthesis and signaling in CK, ABA and (ABA+1-MCP)-treated fruits at different ripening stages. RT-PCR analysis of *SAM1* (A), *SAM3* (B), *ACS1* (C), *ACS2* (D), *ACO1* (E), *ACO4;1* (F), *ACO4;2* (G), *RTE1* (H), *ETR2* (I), *EBP* (J), *ERF4;1* (K) and *ERF4;2* (L) were conducted in CK vs ABA and CK vs ABA+1-MCP treatment. Other details are the same as in Fig 5.

In contrast to the fruits treated with ABA alone, the transcription of the genes *LeZEP*, *NCED4* homologue and *LeNCED2* were considerably up-regulated by ABA+1-MCP over the entire period of ripening (Fig 8A–8C). With respect to ABA catabolism, the expression of *LeCYP707A1* was induced in ABA+1-MCP group (Fig 8D), whereas significant repression was found in *UGT73B3* paralog after the co-application of 1-MCP (Fig 8E). Regarding to the expression of *PYL9*, the continuous up-regulation by exogenous ABA was substantially weakened upon the treatment with 1-MCP (Fig 8F). However, *HAI2* was highly expressed in ABA+1-MCP group throughout fruit ripening, contrary to the down-regulation by ABA treatment (Fig 8G). As an effective inhibitor of ethylene, 1-MCP remarkably suppressed all the representative genes involved in ethylene biosynthesis at all ripening stages with exception of *LeSAM1* and *SAM3* homologue (Fig 9A–9G). Furthermore, expressions of *LeETR6* and *LeERF2* which were involved in ethylene signaling remained at consistent low levels after 1-MCP treatment (Fig 9I and 9J). However, the expression of *ERF4* homologue was observed to be repressed by ABA treatment and up-regulated by ABA+1-MCP over the entire period of ripening (Fig 9K). Compared with ABA treatment alone, however, increased expression of *LeGR* was observed in ABA+1-MCP treated fruits during ripening process (Fig 9H, 9K and 9L).

### RNA-seq analysis of ripening-related TFs under exogenous ABA/NDGA treatments

Recent efforts have identified some key TFs for the positive regulation of tomato ripening, including MADS-RIN[79], TAGL1 [80], FUL1 [81], FUL2 [81], CNR [82], NOR [13] and HB-1[83]. In our study, the expression of *LeFUL1* was significantly elevated by exogenous ABA, and moderately down-regulated by 1.93 fold with NDGA treatment (S8 Table). MADS-RIN is considered to be an essential regulator of tomato fruit ripening [79], which displayed a slightly up-regulated expression in ABA-treated fruits (1.79 fold), and reduced from 671.87 to 396.67 FPKM by NDGA (S8 Table). Similar patterns of altered expression were also observed in the genes encoding TAGL1, FUL2, CNR and NOR under the different exposures (S8 Table). However, *LeHB-1* was expressed at a level 1.48 fold lower in ABA treatment, which was different from the above TF genes (S8 Table). On the other hand, MADS1 has been reported to impact fruit ripening as repressive modulators [84]. Our data showed that the transcription of *MADS1* was considerably down-regulated by exogenous ABA and did not differ in NDGA treatment (S8 Table).

### qRT-PCR validation of the changes in gene expression from RNA-seq data

In order to confirm the accuracy of RNA-seq results, a total of 35 genes with different expression patterns were selected for qRT-PCR validation, including the 19 representative genes involved in the biosynthesis and signaling of ethylene and ABA for time-course RT-PCR analysis mentioned above. In comparison with CK, the expression of all tested genes in ABA and NDGA-treated fruits revealed similar trends between RNA-seq and qRT–PCR, respectively (S9 Table). Furthermore, the scatterplots performed in our study demonstrated a positive correlation between the log2 fold change determined by RNA-seq and RT-PCR (R^2^ = 0.81 in CK vs ABA; R^2^ = 0.71 in CK vs NDGA), thereby verifying the high reliability of the data obtained from deep sequencing (S3 Fig).

## Discussion

In our study, a combination of exogenous treatments with endogenous quantifications may provide an integrated representation of hormones (ABA and ethylene) cellular roles in the regulation of fruit ripening. In agreement with mounting prior studies, our data showed that application of ABA to mature green fruits accelerated ripening progress, concomitant with enhancing ABA content and promoting ethylene synthesis, while they were all inhibited by the treatment of NDGA (Fig 1A–1C) [2,3,5,21,85]. Additionally, the changes in pattern of ACC content, ACS and ACO activity were all in accordance with ethylene evolution (Fig 1D–1F), which was consistent with the previous researches [2,5]. Besides, our result confirmed numerous studies reporting that the maximum ABA content preceded the climacteric rise of ethylene production (Fig 1B and 1C), indicating ABA might act as an upstream regulator of ethylene synthesis and response in fruit ripening [2,3,86,87]. Despite the antagonism between ABA and ethylene observed before endogenous ABA reaches its peak level [16], the ABA inhibition on ethylene synthesis would be gradually weakened as ABA elevated and the increasing ABA to a certain level even could in turn promote the transformation of ACC to ethylene [18–20]. Our analysis consistently suggested that the ethylene production can be stimulated by significantly increased ABA (because of the exogenous ABA treatment), which may be through the induction of the activities of ACO and ACC enzymes as well as ACC content. In contrast, the application of NDGA significantly blocked the accumulation of ABA and consistently keep ABA at a quite low level for a very long time (Fig 1B), which may consequently lead to a delayed triggering of ethylene synthesis. However, it has been reported that a higher level of ethylene production was detected in ABA deficient mutants and transgenic fruits [16, 27]. With respect to this contradiction, we speculated that it may be probably due to the different inhibition mechanisms of ABA between NDGA treatment and ABA genetic deficiency. Taken together, all the biochemical evidences in the present study suggested that the regulatory role of ABA in fruit ripening is at least partially through directly influencing ethylene biosynthesis.

To describe the mechanisms of ABA effects on ethylene at the molecular level, it was essential to investigate the expression of key components involved in the metabolism and signaling of ABA itself and ethylene, respectively. In this study, we identified the DEGs obtained from RNA-seq as the representative genes in response to ABA, and such responses were verified with a time-course analysis by RT-PCR. In plants, the ABA content is modulated by the dynamic balance between biosynthesis and catabaolism [88]. With respect to ABA biosynthesis, the inverse correlation between *ZEP* expression and ABA content may be indicative of a negative feedback regulation exerted by the ABA levels on this gene. Sun et al. have reported that the transcription level of *LeNCED2* was high at the immature stage and declined continually through the remaining stages of fruit ripening, implying the *NCED*s may be involved in the initiation of ABA biosynthesis at the onset of fruit ripening [7,16]. Consistent with prior findings [7,16,50], the expression of *LeNCED2* and *NCED4* homologue were significantly induced/repressed by exogenous ABA/NDGA at the early stage of maturation (Day 3 to 6), which may consequently influence the ABA content. On the other hand, *LeNCED2* transcription has been reported to decrease as fruits ripened [7,16], so the reduced expression of these two *NCED*s in ABA treatment at other time points of later ripening stage was possibly due to the accelerated ripening progress of ABA-treated fruits. Intriguingly, *LeCYP707A1* and *UGT73B3* which were involved in different pathways of ABA inactivation exhibited divergent expression patterns in response to exogenous ABA/NDGA treatments (Fig 5D and 5E). Previous researches have reported that the expression of *LeCYP707A1* would maintain at a quite low level throughout fruit ripening, and its expression was significantly down-regulated under ABA application [16,50]. In our data, the inhibited expression of *LeCYP707A1* in ABA-treated fruits and the reverse in NDGA treatment were in accordance with the reported studies, suggesting the expression of *LeCYP707A1* was opposite to the trend of ABA content during fruit ripening. However, high positive correlations between *UGT73B3* expression and ABA concentration may imply that high level of ABA can promote the formation of ABA conjugates, which was also consistent with the earlier report [17]. With regard to ABA response, our results implied that ABA may positively modulated its signal transduction by up-regulating the receptor *PYL9* and suppress the negative regulator *HAI2*. In all, these results shed light on the molecular mechanism that how ABA regulated its own metabolism and signal transduction.

Many studies have previously reported that ABA may play a positive role in regulating ethylene action during fruit ripening [2,3,17,21,89]. Combining RNA-seq with RT-PCR analysis, our study may provide crucial molecular insights into the key genes affected by ABA in the pathway of ethylene biosynthesis and signaling (Figs 4 and 6). The expression of *SAM3* homologue was significantly induced with exogenous ABA and reached the peak at the 3^rd^ day, then sharply decreased during the later stages of ripening (Fig 6B). This result suggested that the expression of *SAM3* may be up-regulated by exogenous ABA at the early stage of ripening, which probably contributed to the promotion of ethylene production in ABA treated sample. It has been reported that the expression of *LeACS2*, *LeACS4* and *LeACO1* were dominant for ethylene autocatalysis [90,91]. In our data, the transcription of these genes were all significantly up-regulated by exogenous ABA and inhibited by NDGA, providing evidence to support the finding that ABA promoted ethylene synthesis probably via induction of *LeACS2*, *LeACS4* and *LeACO1* [2,14]. In ethylene signaling pathway, RTE1/GR functions as a negative regulator to inhibit the downstream response, whose expression were considerably repressed by ABA and elevated by NDGA treatment during the ripening course (Fig 6H). From this result, it can be proposed that ABA may play an important role in activating ethylene signaling possibly through negatively regulating *LeGR* transcription. *LeETR6*, one of the most important ethylene receptors, appears to be highly expressed at the onset of fruit ripening by ABA as the consequence of climacteric increased ethylene but subsequently decreased from the 9^th^ day [74,92]. Therefore, the expression patterns of *LeETR6* at the early ripening stage could be attributed to the accelerated/delayed climacteric rise of ethylene by exogenous ABA/NDGA. Then, as the negative regulator, the decreased expression of *LeETR6* at the later ripening may positively influence the ethylene response in ABA-treated fruits. However, it seems paradoxical that the receptors which are negative regulators of ethylene response can be transitorily induced by ethylene at the onset of ripening transcriptionally. This result could be explained by the opinion that ethylene can also cause receptor protein degradation by 26S proteasome-dependent pathway, which keeps the actual levels of receptor proteins at a quite low level to activate ethylene signaling [74,92]. Since the *LeERF2* transcript has been reported as ripening-related and not found to be ethylene responsive [77,78], the expression patterns of *LeERF2* in our data was interpreted to be a possible result of accelerated/delayed ripening progress directly affected by the ABA concentration. As *ERF4* homologue was suppressed by ABA in expression and induced by NDGA (Fig 9K), it can be assumed that the *ERF4* homologue possibly play a negative role in the synergetic interaction between ABA and ethylene, which still need more in-depth explorations.

Comparisons between ABA and ABA+1-MCP treatment have been previously conducted by Zhang et al. to investigate the role of ABA and ethylene in the later ripening of fruit [2]. To ensure ABA was fully biosynthesized by exogenous ABA stimulation, 1-MCP was applied 4 days after ABA treatment in ABA+1-MCP group, which was for the characterization of ABA effects on fruit ripening without ethylene response [2,3]. However, different from the experimental design mentioned above, fruits of ABA+1-MCP group in our study were treated with 1-MCP immediately after ABA exposure. Our intention was to explore the interactive mechanism of these two hormones since the onset stage of fruit ripening. Similar to the phenomenon observed in banana [21], the ABA induced tomato ripening was not found in the fruits with subsequent 1-MCP treatment (Fig 7A), indicating ABA’s stimulation of ripening progress was at least partially dependent on ethylene. It has been reported that the application of 1-MCP alone can strongly suppress ethylene production by down-regulating *LeACS*s and *LeACO*s expression [93,94], and block ethylene signaling by preventing ethylene-induced receptor transcriptional increase [74]. Despite of the pretreatment with ABA, the ethylene production and response in our study were still dominantly repressed by the concomitant application of 1-MCP, which was through the negative regulation of the key genes involved in ethylene synthesis and signaling (Figs 7C and 9). Since the significant inhibition of ethylene action in ABA+1-MCP treatment at the beginning, the positive impacts of ABA on its own synthesis and signaling were in nullification until the ceasing of 1-MCP effectiveness at the later stage (Figs 7B and 8). Therefore, this result appeared to support the opinion of Deluc et al. that ethylene may play an essential role in triggering ABA biosynthesis and signaling even at the early stage of ripening [95]. However, the transcription levels of *NCED4* homologue and *LeNCED2* in ABA+1-MCP treatment were observed significantly higher when compared with the fruits treated with ABA alone (Fig 8B and 8C). As for these intriguing results, it can be hypothesized that there probably exist positive feedback regulation on these *NCED*s expression when ABA content as well as other critical genes related to ABA metabolism and signaling were inhibited in ABA+1-MCP treatment. Moreover, the expression abundance of *NCED*s present in the article merely presented the regulatory mechanism at transcriptional level, and the consequent function of NCED proteins would be also regulated by other various factors at post-transcriptional and translational level, which will need more in-depth explorations in the future. Overall, the effects of 1-MCP on ABA-pretreated fruits may illustrate that ABA regulates fruit ripening probably through an ethylene-mediated pathway. It also exhibited the molecular regulatory mechanism of ethylene on ABA synthesis and response at the onset of fruit ripening.

It has been widely accepted that tomato ripening is regulated by plant hormones in conjunction with numerous transcription factors [96]. Moreover, TFs’ involvement in the interaction between hormones was also explored in previous study [12]. Therefore, some specific TF genes were further analyzed in our study for a better understanding of the reciprocity mechanism between ABA and ethylene in the regulation of fruit ripening. MADS-RIN is upstream of ethylene in the regulatory cascade, which functions as a trigger of initial ethylene production and further induces the ethylene responsive genes [97,98]. Dependent upon the presence of a functional *CNR* gene, MADS-RIN can directly target the promoters of many genes involved in the pathways of ethylene biosynthesis and signaling, including *LeACS2*, *LeACS4*, *NR*, *E4* and *E8* [79,99]. In addition, the RIN-mediated ripening regulation can also be activated by ethylene in a positive feedback loop, leading to a remarkably increased expression of *RIN* and subsequent rise in ethylene level [79,98]. Some other ripening regulators, TAGL1 and NOR, are also associated with ethylene regulation, which interact with the promoter of *LeACS2* and *LeACO1*, respectively [13,80]. In the present study, the expression of *MADS-RIN*, *CNR*, *TAGL1* and *NOR* were all elevated by exogenous ABA, and suppressed when endogenous ABA was inhibited by NDGA (S8 Table). As direct targets of the above TFs, the expression of *LeACS2*, *LeACS4*, *LeACO1*, *E4* and *E8* were observed to be significantly up-regulated in ABA treatment and repressed by NDGA (S7 Table). These results suggested that ABA played a promotive role in ethylene biosynthesis and response possibly via the positive regulation of these critical TFs, which consequently contributed to fruit ripening. Also serving as an activator of *LeACO1* transcription, however, *LeHB-1* showed the opposite expression trends compared with the above TFs (S8 Table), which was consistent with the results of Martel et al. [79]. It has been reported that the mRNA of *LeHB-1* is highly accumulated at immature stage but declines to a relatively low level during fruit ripening [83]. Besides, RIN functions as a positive regulator of ethylene response partially by up-regulating *NOR* and *CNR* as well as down-regulating *LeHB-1* [98]. Therefore, the decreased *LeHB-1* expression in ABA treatment was interpreted to be a possible result of accelerated ripening progress induced by ABA. In contrast with the TFs mentioned above, FUL1 facilitate fruit ripening without regulating genes expression related to ethylene biosynthesis or sensitivity [81,100]. In our analysis, *LeFUL1* was significantly induced by ABA and depressed by NDGA, which implied that ABA might regulate *LeFUL1* to mediate ripening in an ethylene-independent manner. On the other hand, MADS1 affects fruit ripening as an inhibitor by weakening MADS-RIN activity, which further repressed the genes of ethylene production and response [84]. From our results, we proposed that ABA improved ethylene action and fruit ripening probably by negatively regulating the expression of *MADS1*.

## Conclusions

**Fig 10:**
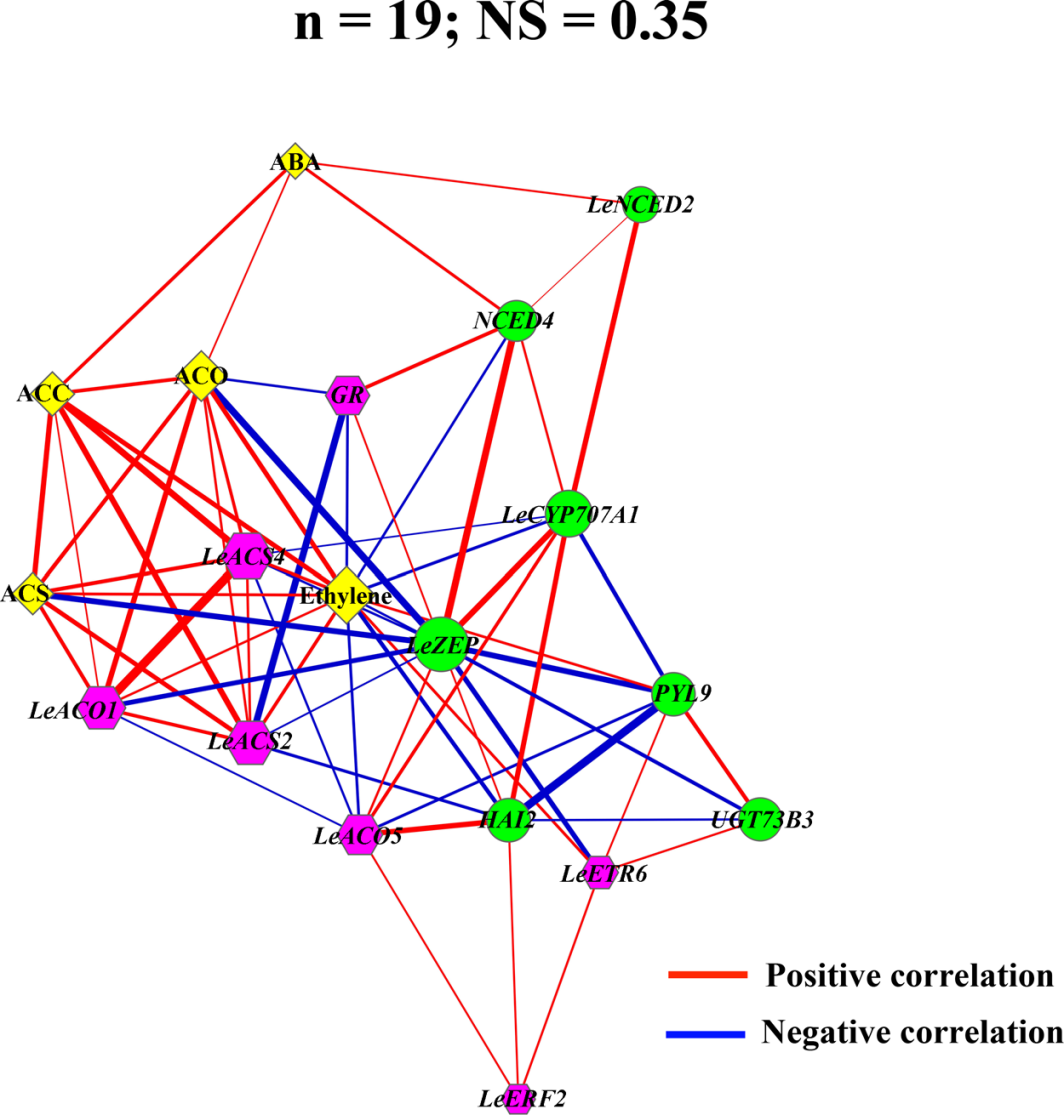
Correlation networks of transcripts and metabolites related to ABA and ethylene. The network diagram is visualized as “organic” layout, with different node shapes representing ABA-related genes (green ellipses), ethylene-related genes (pink hexagons) and metabolites (yellow diamonds). Edges joining the nodes correspond to correlations (| ρ | ≥ 0.40), and positive (ρ > 0) as well as negative (ρ < 0) correlations are shown in red and blue, respectively. Edge thickness is proportional to the| ρ |, while node sizes are proportional to node strengths (ns) which were shown in S11 Table. The number of nodes (n) and network strength (NS) are shown on top of the network.

Taken as a whole, our data suggested that ABA may act as an upstream regulator to modulate ethylene synthesis and signal transduction, which consequently influenced the ripening process. This comprehensive survey not only demonstrated how ABA regulated itself at the molecular level, but also indicated that the increased ABA level may have a positive impact on ethylene production and action by regulating key genes such as *LeACS2*, *LeACS4*, *LeACO1*, *LeGR* and *LeETR6*. Besides, our results also revealed that ethylene might be of great importance to induce ABA accumulation and response at the onset of ripening. By integrating the transcript-metabolite data from crucial time points during ripening, we also conducted a correlation network analysis which may support the idea that ABA may function as a trigger to positively regulate ethylene, and ethylene might appear as the “hub” to play a central regulatory role in the whole network (Fig 10). Moreover, many ripening related TFs, such as *MADS-RIN*, *TAGL1*, *CNR* and *NOR*, were observed to be affected by ABA, implying that a TF-mediated manner may be involved in the interaction between ABA and ethylene. This study extends our understanding of the mechanism that how ABA-initiated tomato fruit ripening, and illustrates the complex mechanism of reciprocity between ABA and ethylene at the transcription level.

## Supporting Information

S1 Fig Principal Component Analysis (PCA) of genes expression data and the abundances of differentially expressed genes identified in ABA and NDGA-treated fruits. (A) PCA was performed to distinguish the majority of transcriptional variance with different treatments. Plots of these components principals also indicated a strong clustering within sample replicates. (B) The number of DEGs (∣log2 FC∣≥1 and P<0.05) compared with the control fruits. The yellow columns represent the up-regulated DEGs and the blues ones represent the down-regulated DEGs.
(TIF)

S2 Fig Global analysis of transcription factors (TFs) across the three samples. (A) The heatmap showing log2 “relative FPKM value” of 1343 TF genes. (B) Families of differentially expressed TF genes from the comparisons of ABA vs CK and NDGA vs CK. The x-axis represents the amount of differentially expressed TFs. The y-axis indicates the distribution of different TF families. The “a” and “b” indicate ABA vs CK and NDGA vs CK, respectively. The red columns represent the number of up-regulated TF genes and the blue ones represent the down-regulated TFs.
(TIF)

S3 Fig Correlation analysis of the log2 fold change (FC) determined by RT-PCR with that obtained from transcriptome analysis. The RT-PCR was performed to quantify the 35 selected genes which showed different expression patterns, and the relative expression changes (FC) were transformed to the log2 scale. Each point in the scatterplot represents the RNA-seq log2 (FC) (x-axis) against the RT-PCR log2 (FC) (y-axis). (A) ABA vs CK. (B) NDGA vs CK.
(TIF)

S1 Table Statistics of RNA-seq alignment in CK, ABA and NDGA-treated samples. (XLS)

S2 Table The transcriptional abundance of genes expressed in CK, ABA and NDGA treated samples. (XLS)

S3 Table Comparison of genes expression between CK and ABA treated samples. The genes’ expression levels were normalized with the value in FPKM. Genes that differed by less than 20% (│log_2_ FC│< 0.25) were assumed to not change in expression level. Genes with P value < 0.05 and │log_2_ FC│≥ 1 were identified as significantly differentially-expressed genes (DEGs). Others were considered as slightly changed.
(XLS)

S4 Table Comparison of genes expression between CK and NDGA treated samples. Other details are the same as the S3 Table.
(XLS)

S5 Table Analysis of transcription factors in CK, ABA and NDGA treated samples. Other details are the same as the S3 Table.
(XLS)

S6 Table Analysis of ABA-related genes in CK, ABA and NDGA treated samples. Other details are the same as the S3 Table.
(XLS)

S7 Table Analysis of ethylene-related genes in CK, ABA and NDGA treated samples. Other details are the same as the S3 Table.
(XLS)

S8 Table Analysis of some specific transcription factors relevant to fruit ripening in CK, ABA and NDGA treated samples. Other details are the same as the S3 Table.
(XLS)

S9 Table Comparison of the genes expression in the Day 9 fruits between qRT-PCR and RNA-seq. The untreated sample (CK) at Day 9 was set as the calibrator for relative expression level, and the relative expression data are presented as means ± SE of three replicates.
(XLS)

S10 Table The full name for all abbreviations appeared in the manuscript. (DOC)

S11 Table Nodes strength (ns) of the network shown in Fig 10. (XLS)
