# Supplementary figures and images for: Comprehensive Analysis of ABA Effects on Ethylene Biosynthesis and Signaling during Tomato Fruit Ripening
Source: PLoS One. 2016 Apr 21;11(4):e0154072. doi: 10.1371/journal.pone.0154072 (PMC4839774; doi:10.1371/journal.pone.0154072)

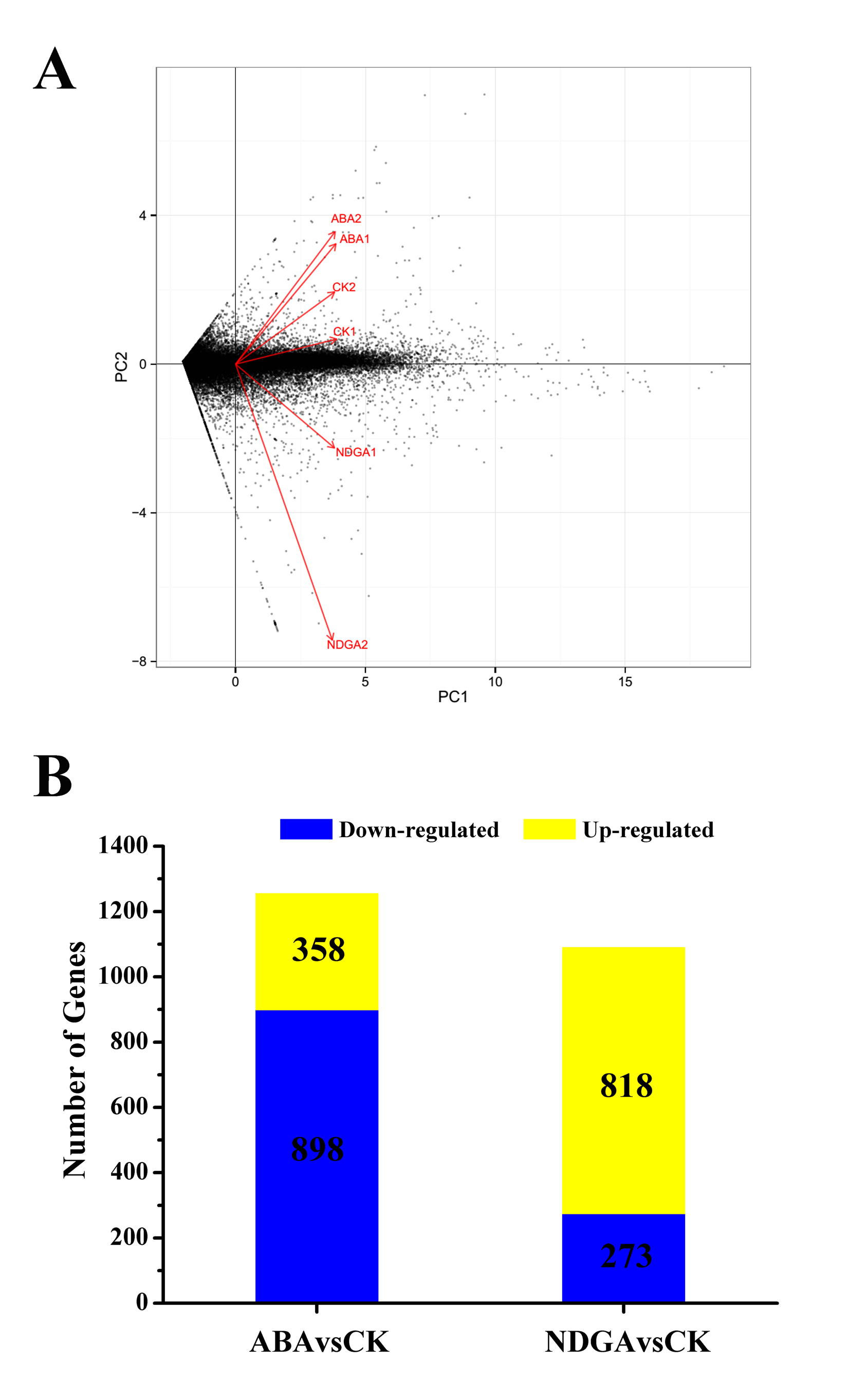

Supplement: S1 Fig — (A) PCA was performed to distinguish the majority of transcriptional variance with different treatments. Plots of these components principals also indicated a strong clustering within sample replicates. (B) The number of DEGs (∣log2 FC∣≥1 and P<0.05) compared with the control fruits. The yellow columns represent the up-regulated DEGs and the blues ones represent the down-regulated DEGs. (TIF) [file pone.0154072.s001.tif]

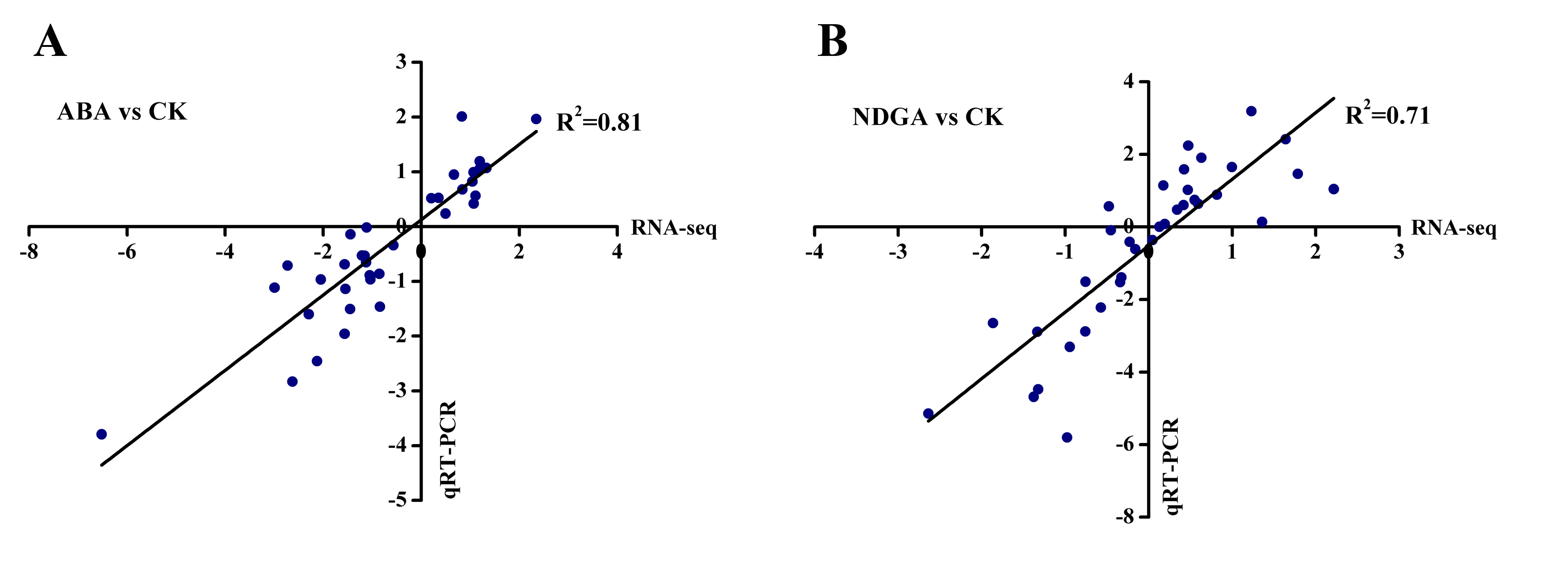

Supplement: S3 Fig — The RT-PCR was performed to quantify the 35 selected genes which showed different expression patterns, and the relative expression changes (FC) were transformed to the log2 scale. Each point in the scatterplot represents the RNA-seq log2 (FC) (x-axis) against the RT-PCR log2 (FC) (y-axis). (A) ABA vs CK. (B) NDGA vs CK. (TIF) [file pone.0154072.s003.tif]
