# Supplementary material for: Comprehensive Analysis of ABA Effects on Ethylene Biosynthesis and Signaling during Tomato Fruit Ripening
Source: PLoS One. 2016 Apr 21;11(4):e0154072. doi: 10.1371/journal.pone.0154072 (PMC4839774; doi:10.1371/journal.pone.0154072)
Supplement: S10 Table — (DOC) [file pone.0154072.s013.doc]

**Table S10 . The full name of genes’ abbreviations presented in the manuscript.**

| **Abbreviations** | **Full name** |
| --- | --- |
| 1-MCP | 1-methylcyclopropene |
| AAO3 | Abscisic aldehyde oxidase 3 |
| ABA8OX | Abscisic acid 8’-hydroxylase |
| ABA-GE | ABA-glucose ester |
| ABA-GS | ABA-glucose ether |
| ABF/AREB | ABRE binding factor/ABRE binding protein |
| ABRE | ABA-responsive elements |
| ACC | 1-aminocyclopropane-1-carboxylate |
| ACO | 1-aminocyclopropane-1-carboxylate oxidase |
| ACS | 1-aminocyclopropane-1-carboxylate synthase |
| AP2a | APETALA2a |
| CNR | Colorless non-ripening |
| CTR1 | Constitutive triple response 1 |
| CYP707A | Cytochrome P450 monooxygenase, subfamily A |
| EBF | EIN3-binding F-box |
| EIN2 | Ethylene insensitive 2 |
| EIN3/EIL | Ethylene insensitive 3/Ethylene-like |
| EIN4 | Ethylene insensitive 4 |
| EIN5/XRN4 | Ethylene insensitive 5/5'-3' exoribonuclease 4 |
| ERF/EBP | Ethylene response factor/ Ethylene-responsive element binding protein |
| ERS2 | Ethylene response sensor |
| ETP1/2 | EIN2-targeting protein 1/ 2 |
| ETR1 | Ethylene response 1 |
| ETR2 | Ethylene response 2 |
| HB-1 | HD-zip homeodomain protein1 |
| MAPK | Mitogen-activated protein kinase |
| MAPKK | Mitogen-activated protein kinase kinase |
| MAPKKK | Mitogen-activated protein kinase kinase kinase |
| MoCo | Molybdenum cofactor |
| NCED | 9-cis-epoxycarotenoid dioxygenase |
| NDGA | Nordihydroguaiaretic acid |
| NOR | Non-ripening |
| NSY | Neoxanthin synthase |
| PP2C | Protein phosphatase 2C proteins |
| PYR/PYL/RCAR | Pyrabactin resistant/Pyrabactin resistant-like/Regulatory component of ABA receptor |
| RAN1 | Responsive-to antagonist 1 |
| RIN | Ripening-inhibitor |
| RTE1 | Reversion-to ethylene sensitivity1 |
| SAM | S-adenosylmethionine |
| SAMS | S-adenosylmethionine synthetase |
| SDR | Short-Chain Dehydrogenase/Reductase |
| SnRK2 | Sucrose non-fermentation kinase 2 protein |
| TAGL1 | Tomato AGAMOUS-LIKE1 |
| UGT | UDP- glycosyltransferase |
| XHT | Xanthoxin |
| ZEP | Zeaxanthin epoxidase |
